# Latent Markov Latent Trait Analysis for Exploring Measurement Model Changes in Intensive Longitudinal Data

**DOI:** 10.1177/0163278720976762

**Published:** 2020-12-11

**Authors:** Leonie V. D. E. Vogelsmeier, Jeroen K. Vermunt, Loes Keijsers, Kim De Roover

**Affiliations:** 1 Department of Methodology and Statistics, 7899 Tilburg University, The Netherlands; 2 Erasmus School of Social and Behavioural Sciences; Department of Psychology, Education & Child Studies/Clinical Child and Family Studies, Erasmus University Rotterdam, The Netherlands

**Keywords:** experience sampling, measurement invariance, latent trait analysis, item response theory, latent Markov modeling

## Abstract

Drawing inferences about dynamics of psychological constructs from intensive longitudinal data requires the measurement model (MM)—indicating how items relate to constructs—to be invariant across subjects and time-points. When assessing subjects in their daily life, however, there may be multiple MMs, for instance, because subjects differ in their item interpretation or because the response style of (some) subjects changes over time. The recently proposed “latent Markov factor analysis” (LMFA) evaluates (violations of) measurement invariance by classifying observations into latent “states” according to the MM underlying these observations such that MMs differ between states but are invariant within one state. However, LMFA is limited to normally distributed continuous data and estimates may be inaccurate when applying the method to ordinal data (e.g., from Likert items) with skewed responses or few response categories. To enable researchers and health professionals with ordinal data to evaluate measurement invariance, we present “latent Markov latent trait analysis” (LMLTA), which builds upon LMFA but treats responses as ordinal. Our application shows differences in MMs of adolescents’ affective well-being in different social contexts, highlighting the importance of studying measurement invariance for drawing accurate inferences for psychological science and practice and for further understanding dynamics of psychological constructs.

## Introduction

Intensive longitudinal data (ILD; e.g., Hamaker & Wichers, 2017) allow one to investigate the dynamics over time of latent (i.e., unobservable) psychological constructs. By frequently gathering data (say at more than 50 measurement occasions) of multiple subjects, new insights regarding subject-specific dynamics can be obtained, which have clinical implications. For instance, studies are being conducted on dynamics in emotions and behaviors related to mental health (e.g., Myin-Germeys et al., 2018; Snippe et al., 2016), and ILD can also be used to tailor interventions to the subject’s real-time dynamics of negative affect (van Roekel et al., 2017). Such data is efficiently gathered by means of Experience Sampling Methodology (ESM; Scollon et al., 2003), in which subjects repeatedly rate questionnaire items over several weeks, say five times a day, at randomized time-points. The recent steep increase in such datasets (e.g., Hamaker & Wichers, 2017; van Roekel et al., 2019) is related to novel technologies to efficiently gather these data with the use of smartphone apps. Hence, there is an urgent need to also develop novel analytical methods.

In order to draw valid inferences about the measured constructs, either for scientific or clinical purposes, it is crucial that the measurement model (MM) is invariant (i.e., constant) across time and subjects (i.e., having within- and between-person invariance). The MM indicates to what extent the latent constructs (or “factors”) are measured by which items, as indicated by the “factor loadings.” For continuous data, the MM is obtained by factor analysis (FA). If measurement invariance (MI) holds, the constructs are conceptually equal and thus comparable across subjects and over time. Often, MI is not tenable because response styles, substantive changes in item interpretation, or changes in the nature of the measured construct may affect the MM. That is, people may differ from each other in their MMs, for instance, depending on psychopathology, but one subject may also differ over time in its own MM, for instance, depending on the social context in which the questionnaire is filled in. When the non-invariance patterns are undetected or ignored, they cause a potential threat to valid inferences using standard methods for comparing factor means across time and subjects. For instance, changes in subjects’ overall emotional well-being may be (partly) due to changes in how subjects interpret the items. Changes in the MM are also important phenomena in their own right. For instance, detecting MM changes is crucial for valid decisions about treatment allocation over time and such changes may even signal the onset of a mental episode. Consider, for example, a psychologist who measures positive affect (PA) and negative affect (NA) in patients with a bipolar disorder. Patients in manic episodes often encounter high arousal PA such as feeling energetic or excited together with high arousal NA such as being irritated or distracted (American Psychiatric Association, 2013). This might result in a MM with one bipolar “arousal” factor contrasting “low” versus “high” arousal. When patients encounter depressive episodes, PA is generally lower and NA at least somewhat higher (Hamaker et al., 2010), which might correspond to a MM with two separate PA and NA factors or one bipolar “valence of affect” factor. Assessing MI thus allows for more accurate conclusions, but may also open up novel possibilities of early detection of subtle changes in daily functioning.

In order to assess for whom and when a MM applies, Vogelsmeier, Vermunt, van Roekel, and De Roover (2019) developed a novel method called latent Markov factor analysis (LMFA) for tracking and diagnosing MM changes for continuous responses in ILD. LMFA combines a latent Markov model (LMM; Bartolucci et al., 2014; Collins & Lanza, 2010) with mixture FA (McLachlan & Peel, 2000; McNicholas, 2016): The LMM clusters subject- and time-point-specific observations into a few dynamic latent classes or “states” according to the MMs underlying these observations and mixture FA evaluates which MM applies for each state. Thus, every state pertains to a different MM and the MM is invariant within one state. Note that not all MMs may apply to each subject. Some subjects may constantly stay in one state while others may transition between different states. By investigating the state memberships, one can see which subjects and measurements are comparable regarding their underlying MM. Investigating the state-specific MMs offers insights into the underlying dynamics and it also helps researchers make decisions about subsequent analyses. For example, when at least “partial” invariance holds across states (i.e., only a few MM parameters differ; Byrne et al., 1989), researchers could study discrete changes in factor means by repeating the LMLTA analysis, restricting invariant MM parameters to be equal across states, and adding factor means to the model.

The new method has raised awareness of possible MM changes in ILD among fundamental and applied researchers who are now eager to evaluate which MM applies to which subject at which time-point (Horstmann & Ziegler, 2020). However, an important limitation of LMFA is the assumption of having normally distributed continuous response items. This assumption is often violated in ILD. Although continuous items are sometimes used (e.g., participants are asked to give their answer by sliding on the Visual Analog Scale from 0 (“not at all”) to 100 (“very much”), many studies use multiple Likert items with five to seven categories for their assessment. Even though it has been shown that items with five or more categories might be treated as continuous (Dolan, 1994), it becomes problematic if the item response distributions are heavily skewed (e.g., when most responses have a 0 score, which is quite common with less frequent thoughts, emotions, or behaviors). FA is not robust against strong deviations from normality and, therefore, may yield inaccurately estimated parameters (Kappenburg-ten Holt, 2014; Rhemtulla et al., 2012; Vermunt & Magidson, 2005). Note that the same problem generally applies to studies that use ordinal items with less than five categories, although this is less common in ILD data. If the normal approximation is clearly incorrect, a better alternative is to treat the items as ordinal and to specify the probability of responding in a certain category by means of “item response theory” or “latent trait” (LT) models, where “trait” refers to a latent construct in the psychometric literature (Vermunt & Magidson, 2016).

The aim of this paper is to combine the strength of LT models to adequately deal with ordinal data with the strengths of LMFA to trace complex measurement non-invariance patterns in the data. The novel and much-needed latent Markov latent trait analysis (LMLTA) for ordinal data is obtained by replacing the mixture FA by a mixture multidimensional version of Muraki’s (1992) “generalized partial credit model” (GPCM) that treats the responses as ordinal. The second section describes LMLTA and how it compares to LMFA. The third section illustrates the empirical value of LMLTA to detect MM changes in ordinal data on adolescents’ well-being in different social contexts. Finally, the fourth section concludes with some points of discussion and future directions of research.

## Method

### Data Structure

In LMLTA, we assume intensive longitudinal observations that are nested within subjects and we assume multiple Likert and, therefore, ordinal items with response categories ranging, for instance, from 1= “strongly disagree” to 5= “strongly agree.” The latter differs from LMFA, where the items are assumed to be continuous variables. The observations are denoted by $y_{ijt}$ with i=1,…,I referring to subjects, j=1,…,J referring to items, and t=1,…,T referring to time-points. Furthermore, g=1,…,G refers to the item categories and the number of categories *G* is assumed to be constant across items. Finally, the number of time-points *T* typically differs across subjects but, for simplicity, we mostly omit the index *i* in *T_i_*. The observations are collected in the 1×J vectors $y_{it}=\left(y_{i1t},\ldots ,y_{iJt}\right)$ that are collected in the T×J subject-specific data matrices $Y_{i}=\left({y\prime }_{i1},\ldots ,{y\prime }_{iT}\right)\prime$. The data matrices are concatenated in the dataset $Y=\left({Y\prime }_{1},\ldots ,{Y\prime }_{I}\right)\prime$ with $\overset{I}{\underset{i=1}{\sum }}T_{i}$ rows.

### Latent Markov Latent Trait Analysis

In LMLTA, just as in LMFA, a LMM specifies transitions between discrete latent states (e.g., manic and depressive state) characterized by state-specific MMs (e.g., state 1 contains one arousal factor and state 2 two affect factors). A LMM is basically a latent class model (Lazarsfeld & Henry, 1968) and thus a method to find unobserved classes of observations with comparable response patterns. A LMM allows subjects to transition between latent classes over time, which is why the classes are called “states.” To get more insight into what possibly predicts state memberships, one may explore the relation between the state memberships and time-varying or time-constant explanatory variables or “covariates.” For instance, sleep quality and disruptions in the daily routine may increase the probability to transition to a manic state (Hamaker et al., 2010). The state-specific MMs are latent variable models that indicate which latent constructs are measured by which items and to what extent. The choice for the type of latent variable model directly follows from the assumed item response distribution: An LT model for ordinal data is used in LMLTA and a FA model for continuous data is used in LMFA.

The parameters in LMLTA can be estimated with the same approaches as in LMFA, using Latent GOLD (LG; Vermunt & Magidson, 2016) syntax. The first approach is a one-step full information maximum likelihood (FIML) estimation (Vogelsmeier, Vermunt, van Roekel, & De Roover, 2019) and the second approach is a three-step (3S) procedure that splits the estimation of the LMM and the state-specific MMs (Vogelsmeier, Vermunt, Bülow, & De Roover, 2019). The latter approach has advantages, especially regarding model selection with covariates. In the following, we first describe the LMM and then introduce the particular LT model applied in this paper and compare it to the FA model in LMFA. Thereafter, we discuss the two possible estimation procedures and the advantages of the 3S estimation.

#### Latent Markov model

**Figure 1.**
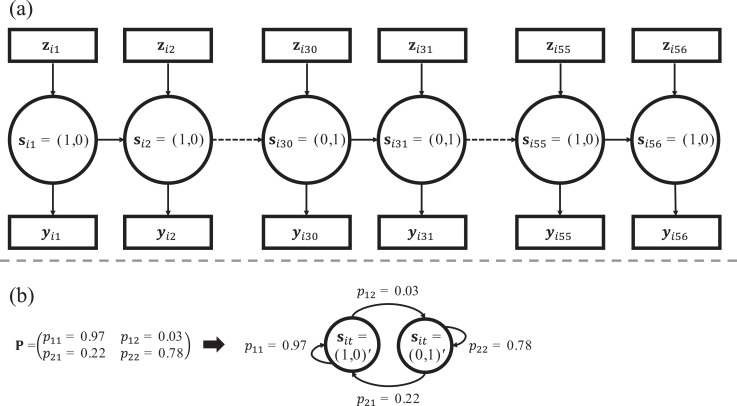
Part (a) is a graphical illustration of a latent Markov chain from the latent Markov latent trait analysis model. The binary vectors $s_{t}={\left(s_{t1}=1,s_{t2}=0\right)}^{'}=\left(1,\;0\right)'$ indicate the state memberships at different time-points *t*, implying that the subject is in state k=1 at time-points 1–29 and 55–56 and in state k=2 at time-points 30–54, implying transitions from state 1 to state 2 at time-point 30 and from state 2 to state 1 at time-point 55. Note that the responses $y_{it}$ are determined by state-specific latent trait measurement models. Furthermore, the covariates $z_{it}$ may influence the state memberships $s_{it}$. Part (b) shows a possible transition probability matrix P for the two states and its corresponding transition diagram that shows how to read the matrix. The diagonal elements correspond to the probabilities to stay in a state and the off-diagonal elements correspond to the transitions away from a state.

The LMM is a probabilistic model with two assumptions (e.g., Bartolucci et al., 2014; Collins & Lanza, 2010): (1) The probability of being in state *k* (with k=1,…,K) at time-point *t* depends only on the state membership at the previous time-point t−1 and not on any other state memberships (first-order Markov assumption) and (2) the responses $y_{it}$ at time-point *t* depend only on the state membership at this time-point (local independence assumption). The sequence of states is called a latent Markov chain (LMC). Figure 1a illustrates a LMC for a single subject: The K×1 vectors $s_{it}=\left(s_{it1},\ldots ,s_{itK}\right)\prime$ contain the binary indicators $s_{itk}$ that are equal to 1 for state *k* and equal to zero for all other states. They determine the state membership at time-point *t*. The U×1 vectors $z_{it}={\left(z_{it1},\ldots ,z_{itU}\right)}^{\prime }$ contain the covariate values $z_{itu}$, with u=1,…,U referring to the subject- and possibly time-point-specific covariates influencing the state memberships. In Figure 1a, state 1 (e.g., the manic state) applies to time-points 1–29 and 55–56, while state 2 (e.g., the depressive state) applies to time-points 30–54.

A LMM is characterized by the “initial state,” “transition,” and “response” probabilities. Together, the parameters form the joint distribution of the observations and states. This is:

1 $$p\left(Y_{i},S_{i}|Z_{i}\right)=p\left(y_{i1},\ldots ,y_{iT,\;}s_{i1},\ldots ,\;s_{iT}|z_{i1},\ldots ,z_{iT}\right)=\overset{\begin{array}{c} initial\;state\\ probabilities \end{array}}{\overset{�}{p\left(s_{i1}\text{|}z_{i1}\right)}}\overset{T}{\underset{t=2}{\prod }}\overset{\begin{array}{c} transition\;\\ probabilities \end{array}}{\overset{�}{p_{\delta_{ti}}\left(s_{it}\text{|}s_{it-1},z_{it}\right)}}\overset{T}{\underset{t=1}{\text{∏}}}\overset{\begin{array}{c} response\;\\ probabilities \end{array}}{\overset{�}{p\left(y_{it}\text{|}s_{it}\right)}}$$

for subject *i*. The initial state and transition probabilities may depend on subject- and time-point-specific covariates $z_{it}$ but, in the following, we will omit an index *z* for simplicity. The initial state probabilities in Equation (1) define the probabilities to start in state k  at time-point t=1 and are collected in a K×1 probability vector π with elements $\pi_{k}=p\left(s_{i1k}=1|z_{i1}\right)$ and $\overset{K}{\underset{k=1}{\sum }}\pi_{k}=1$. In LG, the initial state probabilities are modeled via a logit model as this prevents parameter range restrictions and the covariates also enter through this parameterization as:

2 $$\text{log}\frac{p(s_{i1k}=1|z_{i1})}{p(s_{i11}=1|z_{i1})}=\beta_{0k}+\beta_{k}^{\text{′}}z_{it=1}$$

for  k=2,…,K and with k=1 as the reference category. Here, the initial state intercepts are denoted by $\beta_{0k}$ and the initial state slopes that quantify the effect of the covariates on the initial state memberships are captured by the vectors ${\beta \prime }_{k}=\left(\beta_{k,Z_{i11}},\ldots ,\beta_{k,Z_{i1U}}\right)\prime$.

Transition probabilities are the probabilities to be in state k  at time-point t>1 conditional on state  l (l=1,…,K) at t−1. In a discrete-time (DT-)LMM, intervals between measurements, $\delta_{ti}$, are assumed to be equal. A continuous-time (CT-)LMM (Böckenholt, 2005; Jackson & Sharples, 2002; Vogelsmeier, Vermunt, Böing-Messing, & De Roover, 2019) allows the intervals to differ across time-points and subjects, which is often more realistic in ESM studies and therefore applied throughout the rest of this paper. The transition probabilities $p_{\delta_{ti},lk}=p_{\delta_{ti}}(s_{itk}=1|s_{it-1,l}=1,z_{it})$ are collected in the K×K matrix $P_{\delta_{ti}}$, where the row sums of $P_{\delta_{ti}}$, $\overset{K}{\underset{k=1}{\sum }}p_{\delta_{ti},lk},$ are equal to 1. In a DT-LMM, a multinomial logistic model is used for the transition probabilities:

3 $$\text{log}\frac{p(s_{itk}=1|s_{it-1,l}=1,z_{it})}{p(s_{itl}=1|s_{it-1,l}=1,z_{it})}=\gamma_{0lk}+\gamma_{\prime }^{lk}z_{it}$$

with k≠l, $\gamma_{0lk}$ as transition intercepts, and ${\gamma \prime }_{lk}=\left(\gamma_{lk,Z_{i11}},\ldots ,\gamma_{lk,Z_{i1U}}\right)\prime$ as slopes that quantify the covariate effects on transitioning to another state compared to staying in a state. In Figure 1b, we show how to read a transition probability matrix. The diagonal elements indicate that the probability of staying in state 1 is higher than of staying in state 2. If state 1 is the manic and state 2 the depressive state, we would conclude that the manic state is more persistent for this person.

In the CT-LMM, the transition probabilities themselves are a function of the interval $\delta_{ti}$ and the “transition intensity matrix” Q. The K×K matrix Q contains the transition intensities (or rates) $q_{lk}$ that define the transitions from the origin state *l* to the destination state *k* per very small time unit. For all off-diagonal elements in the matrix Q (i.e., k≠l) the intensities are:

4 $$q_{lk}=\underset{\delta \to 0}{\text{lim}}\frac{p(s_{itk}=1|s_{it-\delta ,l}=1,z_{it})}{\delta }.$$

The diagonal elements are equal to $-\underset{k\neq l}{\sum }q_{lk}$ (Cox & Miller, 1965). The transition probabilities $P_{\delta_{ti}}$ are obtained by taking the matrix exponential of $Q\times \delta_{ti}$. This implies that the probability to transition to another state at two consecutive measurement occasions (i.e., k≠l) becomes increasingly more likely for larger intervals. As can be seen from Equation (4), one may also regress the transition intensities on covariates $z_{it}$ to better understand what may cause the transitions to or away from a state. In the CT-LMM, LG uses a log-linear model for the transition intensities and the covariates are included as follows (again for k≠l):

5 $$\text{log }q_{lk}=\gamma_{0lk}+{\gamma \prime }_{lk}z_{it}.$$

Hence, covariates to predict any of the parameters (i.e., initial state and transition probabilities or intensities) are included by means of regression, as is usually done in LMMs (e.g., Bartolucci et al., 2014; Vermunt et al., 1999; Visser et al., 2009).

Instead of using only observed covariates in any of the parameters, one may also use a time-constant or time-varying latent categorical variable that classifies subjects according to their transition pattern or initial state probabilities into latent classes (Crayen et al., 2017; Vermunt et al., 2008). This “mixture (CT-)LMM” captures the most relevant between-subject differences in the transition process. The number of latent classes can be based on theory and interpretability or selected using information criteria such as the Bayesian information criterion (BIC, Schwarz, 1978) or the convex hull (CHull; Ceulemans & Kiers, 2006) method. An example is shown in the application (Application section).

Finally, the response probabilities $p\left(y_{it}|s_{itk}=1\right)$ indicate the probability for a certain response pattern at time-point *t*, given the state membership at that time-point, $s_{itk}=1$. These response probabilities depend on the state-specific MMs described next.

#### Measurement model

The MMs determine how the responses $y_{itj}$ are defined by the state memberships $s_{itk}=1$. To this end, a latent variable model with state-specific parameters is used in both LMFA and LMLTA. For both methods, it holds that: (1) the responses $y_{itj}$ are indicators of underlying latent factors $f_{it}$, (2) the factors are considered to be normally distributed continuous variables, (3) the responses $y_{ijt}$ are independent given the latent factors, and (4) covariates are only indirectly related to the observed responses via the latent states. As explained before, LMFA and LMLTA differ in the type of latent variable model that is used. In LMFA, the continuous responses $y_{ijt}$ are defined by state-specific linear FA models with parameters that may differ across the latent states. For a single item *j* this is given by (e.g., McLachlan & Peel, 2000):

6 $$E\left(y_{ijt}|f_{it},s_{itk}=1\right)=\;\overset{R_{k}}{\underset{r=1}{\sum }}\lambda_{jrk}\;f_{rit}+\;\nu_{jk},$$

where *R_k_* is the state-specific number of factors, $r=1,\ldots ,R_{k}\;$ indicates a state-specific factor, ${\text{λ}}_{jrk}$ is a state-specific loading of item *j* on factor *r*, $f_{it}=(f_{1it},\ldots ,f_{Rit})\prime$ are subject- and time-point-specific factor scores with $f_{it}∼MVN\left(0,\Phi_{k}\right)$ (note that possible restrictions of $\Phi_{k}$ will be discussed further below), and ${\text{ν}}_{jk}$ indicates a state-specific intercept for item *j*.

In LMLTA, the ordinal responses $y_{ijt}$ are defined by state-specific LT models. It is important to note that there are several LT models that could be used to model Likert-type data (Andrich, 1978; Muraki, 1992; Samejima, 1969). The GPCM (Muraki, 1992) is a relatively flexible and unrestrictive model (Tijmstra et al., 2018) and is therefore considered in this study. More specifically, we use the multidimensional version of the GPCM (e.g., Johnson & Bolt, 2010) and, in order to allow for parameter differences across states, we employ a mixture variant (for previous work on mixture LT models see, e.g., Bolt et al., 2001; Cohen & Bolt, 2005; Rost, 1990; Smit et al., 2000). In contrast to the state-specific FA models in LMFA, the state-specific GPCMs used in LMLTA do not consist of a set of linear models but of a set of adjacent-category (i.e., (g, g +1)) ordinal logit models . More specifically, using as much as possible the same notation as before, the logarithm of the odds of responding in category g+1 instead of responding in category *g* for item *j*, given the factor scores $f_{it}$ and the state membership $s_{itk}=1$ for subject *i* at time-point *t*, has the following linear form:

7 $$\text{log}\left(\frac{p\left(y_{ijtg+1}=1|f_{it},s_{itk}=1\right)}{p\left(y_{ijyg}=1|f_{it},s_{itk}=1\right)}\right)=\overset{R_{k}}{\underset{r=1}{\sum }}\lambda_{jrk}\;f_{rit}+\nu_{jgk}^{\text{*}},$$

for 1≤g≤G−1, with $y_{ijt}=g$ indicating that this response to item *j* is in category *g*. Again, ${\text{λ}}_{jrk}$ is the state-specific loading of item *j* on factor *r*. The $\nu_{jgk}^{*}$ are the G−1  intercepts for each of the adjacent-category log-odds. The logistic model for the probability of response *g* equals:

8 $$p\left(y_{ijt}=g|f_{it},s_{itk}=1\right)=\frac{\text{exp}(\sum_{r=1}^{R_{k}}g\times {\text{λ}}_{jrk}f_{rit}+{\text{ν}}_{jgk})}{\sum_{g\text{′}=1}^{G}\text{exp}\left(\sum_{r=1}^{R_{k}}g\text{′}\times {\text{λ}}_{jrk}f_{rit}+{\text{ν}}_{jg\text{′}k}\right)}.$$

As shown, the loadings are multiplied with the category number and the intercepts are now ${\text{ν}}_{jgk}$, with $\overset{G}{\underset{g=1}{\sum }}{\text{ν}}_{jgk}=0$. The relation between the two sets of intercepts is that ${\text{ν}}_{jgk}^{*}={\text{ν}}_{jg+1,k}-{\text{ν}}_{jgk}$.

When comparing Equation (6) and Equation (7), the loading parameters for the FA model and the GPCM are clearly conceptually similar. In both cases, they indicate how strongly an item *j* measures a latent factor $f_{rit}$ in state *k* (Kankaraš et al., 2011). In contrast, the intercepts are not directly comparable across the two models. In the FA model, there is only one intercept per item and state, ${\text{ν}}_{jk}$, because the responses are treated continuous. For the ordinal responses in the GPCM, there are G−1 free intercept parameters per state, ${\text{ν}}_{jgk}^{*}$.

As in LMFA, the state-specific joint response probabilities for LMLTA at time point *t* are obtained by marginalizing over the latent factors. Moreover, the *J* item responses are assumed to be conditionally independent given the latent factors and the state membership. Therefore, the response probabilities are (e.g., Johnson & Bolt, 2010):

9 $$p\left(y_{it}|s_{itk}=1\right)=\int \ldots \int p\left(f_{it};0,\Phi_{k}\right)\overset{J}{\underset{j=1}{\prod }}p\left(y_{ijt}=g|f_{it},s_{itk}=1\right)df_{it}$$

with $p\left(y_{itj}=g|f_{it},s_{itk}=1\right)$ as in Equation (8) and $p\left(f_{it};0,\Phi_{k}\right)$ denoting the probability density function of the multivariate normal distribution with a mean vector of zero’s and covariance matrices $\Phi_{k}$.

To enable the exploration of all kinds of MM changes, including the number and nature of the factors, an exploratory model is used in both methods. In contrast to a confirmatory model—in which certain factor loadings are assumed to be absent and therefore, set to zero—an exploratory model estimates all loadings.^1^ However, both models are unidentified without further constraints. To partially identify the models and set a scale to the *R_k_* factors, one may restrict the factor means to zero and the factor (co)variances $\Phi_{k}$ to equal an identity matrix, which implies normalized and uncorrelated factors. Alternatively, it is possible to freely estimate the covariance matrix of the factors and instead fix one loading for each of the *R_k_* factors to 1 and one extra loading per estimated correlation to 0 (e.g., for a state with $R_{k}=2$, two loadings would be fixed to 1 and one loading would be fixed to 0). Remaining rotational freedom in the FA model can be dealt with by means of rotation criteria that optimize the simple structure and/or between-state-agreement of the factor loadings (Clarkson & Jennrich, 1988; De Roover & Vermunt, 2019; Kiers, 1997). The identification of the GPCM is more intricate: Despite the model being identified by the constraints imposed so far, one might obtain strongly related parameter estimates and large standard errors. In order to prevent this so-called “empirical underidentification,” $R_{k}-1$ (additional) loadings of different items have to be fixed to 0 in each state (Skrondal & Rabe-Hesketh, 2011).^2^

As becomes apparent from Equation (6) and Equation (7), in either model, the state-specific MMs can differ in terms of the number of factors, the loadings, the intercepts, and the factor covariance matrices. However, there is an important difference between the two methods. In LMFA, states may also differ regarding unique variances, say ${\text{ψ}}_{kj}$, which is variance that is not accounted for by the latent factors. This is possible because the error term in a FA model is assumed to be normally distributed, that is, $e_{ijt}∼N\left(0,{\text{ψ}}_{kj}\right)$. In contrast, in the GPCM, the variance of the error is not a free parameter but fixed to the value of the variance of the standard logistic distribution, $\pi^{2}/3,$ and hence, in LMLTA, also equal across states. Note that, in the GPCM, fixing the error variance is necessary to identify the model (Long, 1997).^3^ Although it might be possible to account for error variance heterogeneity by tailoring “scale adjustment” methods (Magidson & Vermunt, 2007) to LMLTA, this is beyond the scope of this article.

Besides this difference, MI analyses with FA and LT models are similar as their primary concern is to detect parameter differences. However, different words may be used to describe (non-) invariance. When using a LT model, researchers typically specify the lack of invariance, which is called “differential item functioning” (DIF). More specifically, “uniform DIF” is present when only intercepts differ, in our case across latent states, and “non-uniform DIF” is present when loadings differ across states, whether intercepts are equal or not (Bauer, 2017). In contrast, when using a FA model, researchers typically specify which level of invariance has been reached, starting from an invariant number of factors and pattern of zero loadings, followed by invariant loadings, intercepts, and finally unique variances (Meredith, 1993). In the next paragraph, we will describe how to obtain the estimates that are used to investigate the level of invariance in LMLTA.

#### Maximum likelihood estimation

The parameters in LMLTA are obtained with maximum likelihood (ML) estimation. One may choose between (1) the one-step FIML estimation and (2) the 3S estimation, just as is the case for LMFA. However, estimating the LMLTA model with either approach is computationally more complex than estimating the LMFA model. Therefore, LMLTA is limited regarding the number of factors that can be estimated (i.e., including more than three factors is usually unfeasible; see Appendix B for detailed explanations). First, for the FIML estimation (Vogelsmeier, Vermunt, van Roekel, & De Roover, 2019), the following loglikelihood function, derived from the joint distribution in Equation (1), has to be maximized:

10 $$\text{log }L_{FIML}=\overset{I}{\underset{i=1}{\sum }}\text{log}\left(\underset{s_{i1}}{\sum }\ldots \underset{s_{iT}}{\sum }p\left(Y_{i},S_{i}|Z_{i}\right)\right).$$

In LG, the ML estimates are obtained with the forward-backward algorithm (Baum et al., 1970), which is an efficient version of the Expectation Maximization algorithm (Dempster et al., 1977), tailored to LMMs. Additionally, in the Maximization step, a Fisher algorithm is used to update the log-intensities and a combination of the Expectation Maximization and the Newton-Raphson algorithm (De Roover et al., 2017) is used to update the state-specific MM parameters.

Second, the 3S estimation (Vogelsmeier, Vermunt, Bülow, & De Roover, 2019) builds upon Vermunt’s (2010) ML method and decomposes the estimation into three steps. First, in step 1, the state-specific MMs are obtained with a mixture GPCM while treating repeated measures $y_{it}$ as independent. This entails that the relations between the latent states $s_{it}$ at consecutive measurement occasions (i.e., the transitions) and the relations between the state memberships and covariates $z_{it}$ are disregarded. This is valid because observations at one time-point are only indirectly related to covariates and to observations at other time-points, that is, via the latent states. This can also be seen from the graphical representation in Figure 1a.^4^ The mixture GPCM is estimated with a combination of the Expectation Maximization and Newton-Raphson algorithms. Then, in step 2, observations are assigned to the state-specific MMs based on the most likely state membership and the corresponding classification error is calculated. Finally, in step 3, the CT-LMM with covariates is estimated using the state assignments from the previous step as indicators (thus fixing the MMs) while correcting for classification error inherent to the state assignments from step 2. At this point, one may also include a latent class variable to capture differences in transition patterns. The (mixture) CT-LMM model is estimated with a combination of the forward-backward and Newton-Raphson algorithms. Summarized, the steps are:

1. Estimating state-specific MMs (disregarding the dependence of the observations).
2. Assigning observations to the states (depending on the most likely state membership).
3. Estimating the (mixture) CT-LMM with fixed MMs (correcting for step 2’s classification error).

The 3S estimation is almost as good as the FIML estimation in terms of parameter estimation. Only the state recovery is slightly worse and the standard errors can be slightly overestimated (Vogelsmeier, Vermunt, Bülow, & De Roover, 2019).^5^ Apart from that, the 3S approach comes with several advantages. First, step-wise procedures are more intuitive for researchers who use complex methods such as LMLTA or LMFA to analyze their data because it is in line with how they prefer to conduct their analyses (Vermunt, 2010). That is, they see the investigation of the different MMs underlying their data as a first step and the investigation of subject’s transitions between the MMs over time as well as the exploration of possible covariate effects as a next step.

Second, LMLTA (like LMFA) is an exploratory method, which entails that the best number of states *k* and factors per states *R_k_* has to be determined. To this end, a large number of (plausible) models has to be estimated and compared by means of loglikelihood-based criteria that consider fit and parsimony. The evaluation of model selection criteria in LMLTA is beyond the scope of this article but, based on previous findings for related methods (Bulteel et al., 2013; Vogelsmeier, Vermunt, van Roekel, & De Roover, 2019), we suggest to use the BIC in combination with the CHull and compare the three best models in terms of interpretability. Note that CHull balances fit and parsimony without making distributional assumptions and, thus, may perform better for some empirical datasets. In the FIML estimation, the number of models to be compared grows fast. For example, there are nine models when comparing models with one to three states and one to two factors per state. When adding different (sets of) covariates to the CT-LMM, the nine models have to be re-estimated for every set of covariates (e.g., 9×5=45 models for five different sets).^6^ This problem is circumvented in the 3S estimation because the MMs and the CT-LMM are estimated separately. This implies that the model selection can be conducted in the first step, without being concerned about the covariates. Covariates (and latent classes) for the transition probabilities are added when estimating the CT-LMM.^7^ As a result, there would only be 9+5=14 models for five sets of covariates. Note that LG provides Wald tests (Agresti, 1990) that can be used to evaluate whether the covariates are significantly related to the transition or initial state parameters and to determine which MM parameters differ between the states. For the latter, one may also use visual inspection.

Third, the FIML estimation takes several hours for each model while the 3S estimation is usually done in less than 30 minutes. This makes the FIML estimation less desirable, or even unfeasible, when researchers want to explore several covariate effects on MM changes. For all these reasons, we employ the 3S estimation in this study (for details, see Online Supplement S.1).

## Application

### Data

The data stem from a larger “Grumpy or Depressed?” study, which aimed to assess whether daily mood profiles (i.e., variability in affect) would predict the risk for depression in adolescents in the long run as recent work has indicated that the short-term dynamics could be linked to long-term psychopathology (e.g., Maciejewski et al., 2019; for a description of the study, see, e.g., de Haan-Rietdijk et al., 2017; Janssen et al., 2020; van Roekel et al., 2019). Briefly, during three 7-day measurement bursts or “waves” (with approximately 3-month intervals in between), 250 Dutch adolescents (12 to 16 years old) completed up to eight questionnaires per day at random moments (median interval: 2.25 hours).^8^ Out of the 250 adolescents, 164 participated in all three waves, 38 in two of the waves and 48 in one of the waves. In total, the adolescents completed 14,432 questionnaires.

### Measures

For each assessment, adolescents indicated the degree to which 12 affect items applied to them (see Table 1) using 7-point Likert items (ranging from 1= “not feeling the emotion” to 7= “definitely feeling the emotion”). The items covered both PA and NA. The NA items were especially heavily right-skewed. Thus, LMLTA is particularly suited to investigate MM changes. The adolescents also indicated their current social interactions, resulting in the three “social context” covariates “being with friends” (“*fri*”), “being at school/with classmates” (“*cm*”), and “being with family” (“*fam*”), with 0= “no” and 1= “yes.” At the beginning of every ESM wave (i.e., three times), the adolescents completed the Dutch version of the Children’s Depression Inventory (CDI-I; Kovacs, 1992; Timbremont et al., 2008) to screen for (sub)clinical depression (“*dep*”). The 27 items refer to symptoms during the last two weeks scored on three levels representing low severity (0), medium severity (1), and high severity (2); for instance, “I get sad from time to time,” “I get sad often,” and “I’m always sad.” Applying CDI-I cut-off scores (Kovacs, 1992; Timbremont et al., 2008), adolescents with a total score under 12 were categorized as “no depression” (89%) and all others as “(sub-)clinical depression” (11%).

The dataset contains several covariates but, in this study, we focused on the social context and depression as we found these variables particularly interesting to relate to possible MM changes: Emotional experiences may vary depending on the social context. For instance, adolescents may experience elevated positive mood when being among friends, whereas they may be somewhat more irritable and unhappy in the company of their parents, and more demotivated at school (Kendall et al., 2014; Soenens et al., 2017; van Roekel et al., 2013). For some adolescents, mood may be context-independent. Firstly, some adolescents could be in an overall positive mood regardless of the social context (Dietvorst et al., under review). Secondly, adolescents with a depression and those at risk for developing a depression may be rather stable in their emotions in that they often feel unhappy and irritable in any social context (Dietvorst et al., under review; Kendall et al., 2014; Silk et al., 2011). Therefore, for some adolescents, we expect a particular state membership to be more likely in one social context than in another, but also that adolescents differ in their state membership stability, for example, based on their depression level.

### Description of the Applied Mixture CT-LMLTA Model

We will examine the context-dependency of state memberships by regressing the transition intensities (as defined in Equation (5)) on the social context covariates when estimating the CT-LMM (in step 3 of the estimation). To capture potential between-adolescent differences in stability, we will include a latent class variable that automatically classifies the adolescents based on their transition patterns, making the model a mixture CT-LMM as briefly introduced in the Latent Markov Latent Trait Analysis section. To see how many different patterns there are, we will compare models with one to three classes in terms of their fit by means of the BIC and CHull. Note that adolescents are allowed to transition to another class at the beginning of each wave—because subjects may change in their transition patterns over time (possibly related to their wave-specific depression scores—such that the latent class variable is, strictly speaking, another state variable modeled via a DT-LMM (note that a DT model makes sense here as the intervals between the waves are approximately the same for all adolescents). To prevent confusion with the MM state, we will just refer to this latent variable as “class,” with $c_{idv}=1$ referring to being in a particular class *v* (with v=1,…,V) in a particular wave  d (with d=1, 2, 3). To investigate whether experiencing depression affects the class membership, the initial class and transition probabilities of the classes will be regressed on depression.^9^ Moreover, we will evaluate the relation between the social context and the state memberships and investigate whether these relations depend on the class membership. For V>1 and with v=1 as reference category for the class, the specification of the transition intensities of the states (for k≠l) is:

11 $$\text{log}q_{lk}=\gamma_{0lk}+\overset{V}{\underset{v=2}{\sum }}\gamma_{lk,v}c_{itv}+\overset{V}{\underset{v=1}{\sum }}\gamma_{lk,fam,v}\left(fam_{it}\times c_{itv}\right)+\overset{V}{\underset{v=1}{\sum }}\gamma_{lk,cm,v}\left(cm_{it}\times c_{itv}\right)+\overset{V}{\underset{v=1}{\sum }}\gamma_{lk,fri,v}\left(fri_{it}\times c_{itv}\right).$$

The specification of the initial class (for  v=2,…,V) and the transition probabilities for the classes (for v≠b with b=1,…,V) are given by:

$$\text{log}\frac{p(c_{i1v}=1|dep_{i1})}{p(c_{i11}=1|dep_{i1})}=\beta_{0v}+\beta_{v,dep}dep_{id}\text{ and}$$

12 $$\text{log}\frac{p(c_{idv}=1|c_{id-1,b}=1,dep_{id})}{p(c_{idb}=1|c_{id-1,b}=1,dep_{id})}=\gamma_{0bv}+\gamma_{bv,dep}dep_{id},$$

respectively. Note that this application is meant to illustrate the empirical value of tracing MM changes with LMLTA. No hypotheses were pre-registered and all analyses are exploratory so that interesting findings should be validated in future research before drawing any conclusions.

### Obtaining and Investigating the Results of the Mixture CT-LMLTA Model

Below, we follow the three consecutive steps of the 3S estimation described in Latent Markov Latent Trait Analysis section.

#### Step 1 & 2: Estimating state-specific MMs & assigning observations to the states

##### Model selection

To select the best fitting model, we conducted the mixture GPCM analysis for models with one to three states and one to two factors per state (i.e., nine models^10^). Considering one to two factors not only preserves computational feasibility but also makes sense for affect questionnaires as PA and NA are often found as primary affect dimensions that may collapse into one bipolar factor if the emotions are strongly negatively related (Dejonckheere et al., 2018; Vogelsmeier, Vermunt, Bülow, & De Roover, 2019). We selected the model with two states and two factors in each state because it was the best according to the BIC and among the two best models according to the CHull (for model selection details, see the Online Supplement S.2; for the syntax of the selected model, see Online Supplement S.4). Forty-two percentage of the observations belonged to MM 1 and 58% to MM 2.

##### Results and interpretation

To examine the between-state MM differences, we first looked at the state-specific loadings in Table 1. Note that we modeled the covariance matrices in both states. To set the factor scales, we set the loadings of the items “happy” on factor 1 and “unhappy” on factor 2 equal to 1 in both states. To eliminate rotational freedom, we set the remaining loadings of the same items equal to zero. This has led to a well-interpretable simple structure. State 1 is characterized by separate PA and NA factors that correlated negatively (r=−.55) among observations in the same state. This means that adolescents distinguish somewhat between PA and NA, but that adolescents who score high on PA tend to score low on NA and vice versa. In contrast, in state 2, the three low arousal PA (LA-PA) emotions collapse with the NA emotions into one bipolar factor whereas the three high arousal PA (HA-PA) emotions make out the second factor. However, the factors have an even larger negative correlation than in state 1 (r=−.84). This implies that adolescents in state 2 distinguish more between LA-PA and HA-PA than they do between (LA-)PA and NA. Note that strong negative correlations between PA and NA are common in assessments that take place within small time-periods and in questionnaires that contain items with semantic antonyms such as “happy” and “unhappy” or “sad” (Dejonckheere et al., 2018).^11^

**Table 1.** Differences in Factor Loadings, Factor (Co-)Variances, Factor Correlations, and Item Means Across the two States.

	State 1 Loadings ${\text{λ}}_{jr1}$		State 2 Loadings ${\text{λ}}_{jr2}$		Between-State Loading Difference Statistics						Item Means	
	r=1	r=2	r=1	r=2	r=1			r=2			State 1	State 2
Item *j*	PA	NA	HA-PA	LA-PA/NA	Wald	df	p-value	Wald	df	p-value		
relaxed	**0.63**	0.03	0.17	**−0.71**	5.34	1	0.02	7.22	1	< 0.01	5.72	6.89
content	**0.96**	0.00	0.32	**−1.09**	6.65	1	< 0.01	7.31	1	< 0.01	5.76	6.92
confident	**0.46**	0.02	0.21	**−0.48**	1.53	1	0.22	6.93	1	< 0.01	5.66	6.85
happy	**1.00**	0.00	**1.00**	0.00	/	/	/	/	/	/	5.62	6.81
energetic	**0.51**	0.00	**1.18**	0.30	6.67	1	< 0.01	3.21	1	0.07	5.21	6.41
excited	**0.69**	0.00	**1.23**	0.17	6.27	1	0.01	1.35	1	0.25	5.27	6.60
sad	0.04	**0.74**	0.05	**0.88**	0.18	1	0.67	1.44	1	0.23	1.09	1.03
unhappy	0.00	**1.00**	0.00	**1.00**	/	/	/	/	/	/	1.06	1.02
disappointed	0.08	**1.06**	0.14	**1.18**	0.34	1	0.56	0.30	1	0.58	1.07	1.04
angry	0.14	**0.99**	0.14	**1.08**	0.00	1	1.00	0.11	1	0.74	1.04	1.02
nervous	−0.01	**0.41**	0.10	**0.52**	1.70	1	0.19	0.33	1	0.57	1.24	1.09
irritated	0.00	**0.48**	0.05	**0.48**	0.53	1	0.47	0.00	1	1.00	1.24	1.16
Variances (chol)	3.69	3.53	2.18	0.96	14.02	1	< 0.01	23.94	1	< 0.01	/	/
Cov. (chol) with q=1	/	−2.32	/	−1.50	/	/	/	2.38	1	0.12	/	/
Cor. with q=1	/	−0.55	/	−0.84	/	/	/	/	/	/	/	/

*Note.* PA = Positive Affect; NA = Negative Affect; HA = High Arousal; LA = Low Arousal; Cov. = covariances; chol = Cholesky decomposed; Cor. = correlation; *j* refers to items, and *r* to factors. For identification purposes, we set the underlined loadings of the items “happy” on the first factors (r=1) equal to 1 and on the second factors (r=2) equal to 0 and the underlined loadings of the item “unhappy” on the first factors (r=1) equal to 0 and on the second factors (r=2) equal to 1. For each item and state, the loading with the largest absolute value is printed in boldface.

Next, we investigated the between-state differences in the mean item scores. These scores are directly related to the state- and category-specific intercepts (which are given in Supplement 3 Table 2), but the item means are easier to interpret. They are calculated as $\overset{G}{\underset{g=1}{\sum }}g\times p\left(y_{itj}=g|f_{it}=0,s_{itk}=1\right)$ and thus a function of the logistic model for the probability of giving a response *g* as defined in Equation (8) with the factor scores $f_{it}$ set equal to 0=(0, 0)′. As can be seen from Table 1, the means of the PA items are higher than the means of the NA items in both states. However, the PA means are lower in state 1 than in state 2. Thus, adolescents who distinguish more between LA-PA and HA-PA report a slightly better mood.

#### Step 3: Estimating the mixture CT-LMM with fixed MMs

Since each adolescent may have a different MM at different measurement occasions, we examined adolescents’ transitions from one state to another. Additionally, as motivated above, we investigated (1) whether adolescents differed in their state- (and thus MM-) memberships by classifying the adolescents based on their transition patterns (i.e., transitions between states from one measurement occasion to the next) into latent classes that could differ across the three waves, (2) whether the wave-specific covariate depression had an influence on this class membership, and (3) whether the time-varying social context covariates (family, classmates, and friends) affected the transitions between the states and whether these effects differed across classes. To this end, we estimated the mixture CT-LMM with the state assignments from step 2 of our analysis as indicators, while accounting for the inherent classification errors. Note that the correction was hardly necessary as the classification errors were very small due to a high state separation (with $R_{entropy}^{2}=.86$),^12^ which means that most observations were assigned to a state with a high certainty in step 2 of the analysis.

##### Model selection

We first estimated the “full” model as summarized in Equation (11) and (12) for one to three classes (i.e., with all possible covariates as just described). In the two- and three-class solutions, the effects of depression on the initial class ($\beta_{v,dep}$) and on the transition probabilities for the classes ($\gamma_{rv,dep}$) were non-significant. Hence, the class membership was unaffected by the level of depression. Furthermore, the effects of being with family ($\gamma_{lk,fam,v}$) and classmates ($\gamma_{lk,cm,v}$) on the transitions between the states significantly differed across classes, whereas the effect of being with friends ($\gamma_{lk,fri,v}$) did not significantly differ across classes. However, being with friends in itself had a significant effect on the transitions between the states (i.e., there was an effect but it did not differ across classes). Therefore, we re-estimated the two- and three-class models while omitting depression and the conditional effect of being with friends but including a class-independent effect of being with friends (i.e., $\gamma_{lk,fri}$). Comparing all full and “reduced” models, the reduced three-class model had the best fit according to the BIC and was among the best three models according to the CHull (for model selection details, see Online Supplement S.5; for the syntax of the full and reduced three-class models, see Online Supplement S.4).^13^

##### Results and interpretation

Table 2 shows the parameters of the final model. First, we looked at the three classes that captured differences in adolescents’ between-state transitions. To this end, we computed the probabilities for the median interval (2.25 h) and mean covariate values: ^14^

**Table 2.** Parameter Estimates for the Mixture CT-LMM in Step 3 of LMLTA.

	Parameter	Estimate	SE	z-value	p-value	Wald	df	p-value
DT-LMM for Classes								
Initial Class	$\beta_{0v=2}$	0.19	0.22	0.90	0.37	12.12	2	< 0.01
	$\beta_{0v=3}$	0.60	0.19	3.22	< 0.01			
Transition Intercepts	$\gamma_{0b=1,v=2}$	−2.02	0.54	−3.75	< 0.01	103.6	6	< 0.01
	$\gamma_{0b=1,v=3}$	−1.18	0.33	−3.62	< 0.01			
	$\gamma_{0b=2,v=1}$	−1.62	0.49	−3.34	< 0.01			
	$\gamma_{0b=2,v=3}$	−0.70	0.30	−2.35	0.02			
	$\gamma_{0b=3,v=1}$	−2.61	0.43	-6.04	< 0.01			
	$\gamma_{0b=3,v=2}$	−2.86	0.47	−6.06	< 0.01			
CT-LMM for States								
Initial State	$\beta_{0k=2}$	0.02	0.13	0.17	0.86	0.03	1	0.86
Transition	$\gamma_{0l=1,k=2}$	−0.55	0.20	−2.69	< 0.01	23.19	2	< 0.01
Intercepts	$\gamma_{0l=2,k=1}$	−0.08	0.20	−0.40	0.69			
Effect of Class	$\gamma_{l=1,k=2,v=2}$	0.0	0.25	−0.01	0.99	588.60	4	< 0.01
	$\gamma_{l=1,k=2,v=3}$	−7.21	0.38	−19.16	< 0.01			
	$\gamma_{l=2,k=1,v=2}$	−1.71	0.27	−6.32	< 0.01			
	$\gamma_{l=2,k=1,v=3}$	−8.74	0.60	−14.55	< 0.01			
Effect of Family × Class	$\gamma_{l=1,k=2,fam,v=1}$	−0.48	0.22	−2.17	0.03	40.49	6	< 0.01
	$\gamma_{l=1,k=2,fam,v=2}$	−0.10	0.20	−0.51	0.61			
	$\gamma_{l=1,k=2,fam,v=3}$	−1.11	0.55	−2.02	0.04			
	$\gamma_{l=2,k=1,fam,v=1}$	−0.63	0.22	−2.81	< 0.01			
	$\gamma_{l=2,k=1,fam,v=2}$	−1.12	0.26	−4.22	< 0.01			
	$\gamma_{l=2,k=1,fam,v=3}$	−2.27	1.47	−1.54	0.12			
Effect of Classmates × Class	$\gamma_{l=1,k=2,cm,v=1}$	−2.62	0.39	−6.77	< 0.01	113.30	6	< 0.01
	$\gamma_{l=1,k=2,cm,v=2}$	−0.75	0.25	−3.04	< 0.01			
	$\gamma_{l=1,k=2,cm,v=3}$	−2.70	1.87	−1.45	0.15			
	$\gamma_{l=2,k=1,cm,v=1}$	−1.30	0.26	−4.94	< 0.01			
	$\gamma_{l=2,k=1,cm,v=2}$	0.51	0.25	2.07	0.04			
	$\gamma_{l=2,k=1,cm,v=3}$	−0.96	0.84	−1.14	0.25			
Effect of Friends	$\gamma_{l=1,k=2,fri}$	−0.63	0.16	−3.92	< 0.01	16.96	2	< 0.01
	$\gamma_{l=1,k=2,fri}$	−0.39	0.17	−2.36	0.02			

*Note*. DT = discrete-time, CT = continuous-time, LMM = Latent Markov Model, Family (fam) refers to being with family, Classmates (cm) refers to being at school/with classmates, Friends (fri) refers to being with friends, *v* refers to a class in wave *d*, *b* refers to a class in wave d−1, *k* refers to a state at time-point *t*, and *l* refers to a state at time-point t−1. The overall Wald test for the differences in parameters between the classes for Family × Class was Wald (4) =18.29, p < 0.01. For Classmates × Class the Wald test was Wald (4) =27.86, p < 0.01. The covariate effects on the state transitions can be understood as follows: negative estimates imply that the log intensities and therefore also the transition probabilities decrease (e.g., the estimate ${\hat{\gamma }}_{l=2,k=1,fam,v=2}=-1.12$ means that the probability of transitioning from state l=2 to state  k=1 for a subject in class v=2 is lower when the subject is with family compared to when the subject is not with family). The estimates can also be used to calculate the transition probabilities for any class, covariate value and time-interval of interest. An example showing how to calculate the parameters in R is provided in Online Supplement S.6.

12 $$P_{states}^{v=1}=\left(\begin{array}{cc} 0.86 & 0.14\\ 0.44 & 0.56 \end{array}\right),\;P_{states}^{v=2}=\left(\begin{array}{cc} 0.58 & 0.42\\ 0.15 & 0.85 \end{array}\right),\;P_{states}^{v=3}=\left(\begin{array}{cc} 1 & 0\\ 0 & 1 \end{array}\right).$$

Class 1 and 2 each include 25% of the adolescents, whereas 50% were assigned to class 3. As can be seen from the relatively large values in column 1 of $P_{states}^{v=1}$, adolescents in class 1 had a higher probability to transition to and stay in state 1 (i.e., PA vs. NA), whereas adolescents in class 2 had a higher probability to transition to and stay in state 2 (HA-PA vs. LA-PA/NA), which can be seen from the relatively large values in column 2 of $P_{states}^{v=2}$. Thus, 25% of the adolescents are mostly in state 1 and 25% are mostly in state 2. In class 3, transitions to another class were highly unlikely since the (rounded) off-diagonal elements are equal to zero in $P_{states}^{v=3}$, implying that adolescents in this class largely showed within-person invariance. Over the three waves with 3-month intervals, more adolescents transitioned to the stable class 3, as can be seen from the third column of the matrix containing the probabilities to transition between classes from one wave to another:

14 $$P_{classes}=\left(\begin{array}{ccc} 0.69 & 0.09 & 0.21\\ 0.12 & 0.59 & 0.29\\ 0.06 & 0.05 & 0.88 \end{array}\right)\;.$$

Thus, over the three waves, adolescents developed a more stable assessment of their feelings. Perhaps their repeated answers to the questionnaire helped them to develop emotional awareness.

Considering the most prominent results (i.e., p<0.01) of the social context covariates, we can see that the two class-dependent covariates (being with family and with classmates) had no effect in the stable class 3. In class 1 and 2, being with family decreased the probability of moving to state 1 (${\hat{\gamma }}_{l=2,k=1,fam,v=1}=-0.63$; ${\hat{\gamma }}_{l=2,k=1,fam,v=2}=-1.12$). This implies that the probability to be in state 2 increased. Thus, when being with family (compared to not being with family), adolescents distinguish more between LA-PA and HA-PA and less between (LA-)PA and NA. One can imagine that HA-PA and LA-PA can emerge as separate factors. For example, while watching Netflix with the family, adolescents might feel “content” or “relaxed” but not “excited.”

For adolescents in class 1, being with classmates decreased both the probability of moving to state 2 and moving to state 1 (${\hat{\gamma }}_{l=1,k=2,cm,v=1}=-2.62$; ${\hat{\gamma }}_{l=2,k=1,cm,v=1}=-1.30$), such that state memberships became more stable. It is plausible that schools provide a relatively structured and therefore stable environment, which affects adolescents’ emotional well-being less strongly than the more volatile experiences of being with family and friends.

In all three classes, being with friends (compared to not being with friends) decreased the probability of moving to state 2 (${\hat{\gamma }}_{l=1,k=2,fri}=-0.63$).^15^ The same was found for adolescents being with classmates in class 2 (${\hat{\gamma }}_{l=1,k=2,cm,v=2}=-0.75$). This implies that, for them, the probability to be in state 1 increased and thus, that adolescents tended to distinguish more between PA and NA. One possible explanation is that social support of friends is very important for adolescents (Bokhorst et al., 2010), so that adolescents who are “unhappy,” for instance, because they failed a test, may still feel “content” when they are among their friends (and possibly classmates). Although one would expect to find an elevated mood when adolescents are with their friends (Kendall et al., 2014; van Roekel et al., 2013), the PA in this state is slightly lower than in state 2, perhaps because adolescents visit their friends more often when feeling bad and/or are more likely to discuss negative emotions with friends than with, for instance, family.

##### Summary of the LMLTA findings

We conclude that two MMs were underlying adolescents’ responses: in state 1 (42% of all observations), adolescents distinguished mainly between PA and NA and had a slightly worse mood than in state 2 (58% of all observations), where adolescent distinguished more between LA-PA (e.g., content) and HA-PA (e.g., excited) than they did between (LA-)PA and NA; (2) three state-transition patterns were found, implying that adolescents indeed differed in the stability of their emotional experience: in class 1, adolescents frequently transitioned between the states with a high probability to be in state 1; in class 2 they frequently transitioned but were more likely to be in state 2, and in class 3, they mainly stayed in one of the two states; (3) depression did not influence the class membership and thus the transition pattern; (4) for the unstable classes 1 and 2, being with family increased the probability to be in state 1; (5) for class 1, being with classmates increased the probability of staying in either state; (6) for all classes, being with friends—and for class 2, being with classmates—increased the probability to be in state 1. Our results show that researchers can obtain valuable insights from investigating MM changes and that it is important to consider the possibility that changes in positive or negative affect (e.g., evaluated by means of investigating changes in sum scores) could come from variability in the underlying MMs. Therefore, the novel method LMLTA (or LMFA) can improve the emerging trend of studying emotional dynamics as predictors of future well-being and psychopathology. In the future, it would be interesting to study the MMs and transition patterns in a larger group of adolescents with (different levels of) depression and to include other covariates that may explain differences in transition patterns and state-membership probabilities. For example, stress can cause a simplified representation of emotions (Dejonckheere et al., 2019), which can lead to very high correlations between emotions.

## Discussion

In recent years, the awareness of potential measurement model (MM) changes in intensive longitudinal data—and the associated comparability problems—increased among substantive researchers and they are keen to evaluate such changes with new methods like latent Markov factor analysis (LMFA) (Horstmann & Ziegler, 2020). Understanding subject- and context-dependent MMs in more detail may benefit future studies on daily life dynamics and also have clinical implications, for instance, when MMs can be related to the onset of psychopathology. However, up to now, only researchers whose data contained (approximately) normally distributed continuous items could benefit from LMFA, whereas intensive longitudinal data often contain ordinal item responses with few categories or skewed distributions. In this article, we combined the strength of LMFA to evaluate MM changes over time with the strength of latent trait (LT) models accommodate ordinal data in the new latent Markov latent trait analysis (LMLTA).

We showed that LMFA and LMLTA are similar as they both capture discrete changes or differences in subjects’ underlying MM and thus in how latent constructs are measured by observed item responses. The difference lies in the type of latent variable model that is used to specify the relations between the latent constructs and observed variables, which directly follows from the assumed distribution of the observed item responses. Whereas the factor analysis (FA) model in LMFA assumes normally distributed continuous item responses, the generalized partial credit model (GPCM) in LMLTA assumes ordinal responses. The GPCM differs from the FA model in that (1) it has one intercept per item category and not one per item, (2) error variances cannot be freely estimated as they need to be fixed for identification, (3) rotation is only possible by means of setting identifying constraints, and (4) the number of constructs that can be included in the model is limited due to the computationally more complex estimation. This implies that, in LMLTA, more parameters have to be estimated, error variances are assumed to be identical across states, and the model specification is less flexible than in LMFA. For these reasons, we believe that LMFA should be the preferred method if the items are approximately normal and are measured with at least five categories (Dolan, 1994). The robustness of LMFA against violations of normality has never been evaluated, however. In the future, it would therefore be important to formulate more concrete guidelines on the basis of a simulation study that is tailored to intensive longitudinal data and that provides information on the robustness of LMFA, for instance, in terms of sample size and number of measurement occasions, degree of skewness, and number of item response categories. In the meantime, researchers should be cautious and, in case of doubt, opt for LMLTA and compare its results to those of LMFA.

By investigating differences in discrete MM changes over time in relation to covariates, LMLTA is a valuable step toward validly studying psychological dynamics. Additionally, as briefly described in the introduction, the results of LMLTA may also help researchers decide on subsequent analyses. When invariance is clearly untenable, further evaluating dynamics with an approach that builds upon the invariance framework is simply not appropriate. However, observations for which invariance holds can be used to study dynamics in latent processes with standard analyses (e.g., growth models, Muthén, 2002, or dynamic structural equation modeling, Asparouhov et al., 2017), without results being influenced by differences in the underlying MMs. Moreover, if partial invariance holds across states, one may also continue with latent process analyses either by removing items with non-invariant parameters or by allowing for state- (or subject- and time-point-) specific parameters. Finally, we would like to highlight that there is no gold standard yet in how to analyze intensive longitudinal data and the latent variable framework that LMLTA is based on is only one possibility. There are various other reasonable frameworks for analyzing the data (e.g., network psychometrics; Epskamp, 2020; Marsman et al., 2018) and decisions about the data analysis can considerably impact, for example, clinical recommendations (Bastiaansen et al., 2020). Therefore, in the future, it would be desirable to compare perspectives about psychological phenomena from various modeling approaches.

## Supplemental Material

Supplemental Material, Accepted_VogelsmeierEtal2020_LMLTA_highlightedSupplement - Latent Markov Latent Trait Analysis for Exploring Measurement Model Changes in Intensive Longitudinal Data Click here for additional data file. Supplemental Material, Accepted_VogelsmeierEtal2020_LMLTA_highlightedSupplement for Latent Markov Latent Trait Analysis for Exploring Measurement Model Changes in Intensive Longitudinal Data by Leonie V. D. E. Vogelsmeier, Jeroen K. Vermunt, Loes Keijsers and Kim De Roover in Evaluation & the Health Professions

## Appendix A

### List of Abbreviations

**Table:**

3S	Three-step
BIC	Bayesian information criterion
CDI-I	Children’s Depression Inventory
CHull	Convex hull
CT	Continuous-time
DIF	Differential item functioning
DT	Discrete-time
ESM	Experience sampling methodology
FA	Factor analysis
FIML	Full information maximum likelihood
GPCM	Generalized partial credit model
HA	High arousal
ILD	Intensive longitudinal data
LA	Low arousal
LG	Latent GOLD
LMC	Latent Markov chain
LMFA	Latent Markov factor Analysis
LMLTA	Latent Markov latent trait analysis
LMM	Latent Markov model
LT	Latent trait
MI	Measurement invariance
ML	Maximum likelihood
MM	Measurement model
NA	Negative affect
PA	Positive affect

### Appendix B

The main complication in estimating LMLTA is the lack of a closed form expression for the *R_k_*-dimensional integral in the marginal density in Equation (9), $p\left(y_{it}|s_{itk}=1\right)$. This is different in LMFA: As the factors and observations are both normally distributed continuous variables, the marginal density in Equation (9) can be written as multivariate normal distribution with means $\nu_{k}$ and covariance matrices $\Sigma_{k}=\Lambda_{k}\Lambda_{k}^{\prime }+\Psi_{k}$, where $\Lambda_{k}$ is the state-specific $J\;\times \;R_{k}$ loading matrix and $\Psi_{k}$ contains the unique variances ${\text{ψ}}_{kj}$ on the diagonal and zeros on the off-diagonal. In LMLTA, LG approximates the integral using Gauss-Hermite quadrature with *M* quadrature nodes per factor. For instance, with M=10 and $R_{k}=2$, there are $10^{2}$ nodes in total. The integration in Equation (9) is then substituted by *R_k_* summations (Vermunt & Magidson, 2016):

A1 $$p\left(y_{it}|s_{itk}=1\right)=\overset{M}{\underset{m=1}{\sum }}\ldots \overset{M}{\underset{o=1}{\sum }}\left(\overset{J}{\underset{j=1}{\prod }}p\left(y_{ijt}=g|m,\ldots ,o,s_{itk}=1\right)A_{m}\cdots A_{o}\right).$$

Here, m,o=1,…,M indicate the nodes, which are the *M* roots of the *M*th-order Hermite polynomial, and *A_m_* indicates their corresponding weights. The values of the nodes and weights can be found in Abramowitz and Stegun (1970).^16^ Note that usually at least 10 nodes per factor are used (Lesaffre & Spiessens, 2001). As the number of nodes and thus the computational effort increases exponentially, specifying models with more than three factors is often unfeasible.

## Notes

1. If desired, however, a confirmatory model may also be used in both LMFA and LMLTA.

2. Note that these constraints could also be used to solve rotational freedom in the FA model (Vermunt & Magidson, 2016).

3. Note that this is generally a limitation, also in other LT models, and it is often ignored. However, it is important to understand that possible differences in error variances across states will be captured as loading and intercept differences (Long, 1997). For instance, when in one state the error variance is two times larger than in the other state, the loadings and intercepts in that state will be $\sqrt{2}$ times smaller than in the other state.

4. It is important to note, however, that the standard errors of the parameters would be underestimated without applying a correction because observations are nested, and thus dependent, within subjects. This is only necessary when relying on hypothesis tests to determine which parameters differ significantly between the states (the possibility to use such tests will be describe below). By providing LG with a “primary sampling unit” (PSU) identifier, the estimation takes into account that observations may come from the same sampling unit, i.e., the subject (Vermunt & Magidson, 2016).

5. Note that another limitation concerns the possible violation of the first-order Markov assumption (i.e., that the state-membership at time-point *t* is not only influenced by the state-membership at t−1 but also, e.g., by the occupied state at t−2; see Latent Markov Latent Trait Analysis section). Only the FIML approach could capture such a dependency. However, with regard to other violated assumptions (e.g., covariates having direct effects on indicators), the FIML approach would suffer more from bias than the 3S approach but discussing the consequences is beyond the scope of this article (for a description of the problems and solutions, see Vermunt & Magidson, 2020).

6. Note that the number of models grows even faster when also exploring different numbers of latent classes.

7. The MMs are kept fixed (thus, are not re-estimated) once the covariates are included to the CT-LMM. Otherwise, the optimal model complexity in terms of factors and states could change (Di Mari et al., 2016).

8. Note that the researchers studied affect dynamics at multiple time scales because affect can change within hours, days, and weeks; Houben et al., 2015). This measurement burst design (Nesselroade, 1991) enabled the combination of different time scales (i.e., daily fluctuations in affect and long-term change in depression), while minimizing the burden for the participants. Furthermore, random measurement occasions facilitated capturing the continuously evolving daily dynamics in affect, minimizing effects of anticipated beeps and structural day routines on the assessment of affect (van Roekel et al., 2019).

9. Note that some adolescents (17 in wave 1, 26 in wave 2, and 18 in wave 3) missed out on the CDI-I questionnaire, but did participate in the ESM study, and therefore had no depression score in a given wave. For adolescents who had at least one score in any wave, we imputed their average total score and calculated the scale scores according to the cut-off values. For the other cases (i.e., nine in wave 1, one in wave 2, and none in wave 3), LG automatically uses the average effect for predicting the initial class and transition probabilities.

10. The nine models are [2 2 2], [2 2 1], [2 1 1], [1 1 1], [2 2], [2 1], [1 1], [2], and [1]. The notation means, for instance, that model [2 1 1] has three states with 2, 1, and 1 factors in each state, respectively.

11. One might wonder if the loading pattern emerged only because of our chosen identification constraints. Therefore, for the same model, we also investigated a solution without correlations between the latent factors, with variances set to 1, and with the loadings of the item “irritated” set to 0 for the first factor in both states. The results can be found in the Online Supplement S.3. Again, the solution shows that the three HA-PA emotions in state 2 stand out from the other emotions. Thus, we are confident about this finding.

12. The $R_{entropy}^{2}$ value defines how much the state membership prediction improves when using the observations $y_{it}$ compared to when the state membership is predicted without them. The values range from zero, where the prediction is no better than chance, to one, where the prediction is perfect.

13. Note that we also explored whether using the total depression scores instead of the dichotomous cut-off scores would change the results, which was not the case.

14. As previously described, Figure 1 shows how to read a transition probability matrix. In Online Supplement S.6, we provide R code for calculating the transition probability matrix from the parameter estimates in Table 2 for any class, covariate, and time-interval of interest.

15. Note that there is only one effect because the relation between being with friends and the state membership was not conditional on the classes in the final model.

16. Note that the formula in Equation (A1) assumes that the factor scores are uncorrelated. When covariances are non-zero, Cholesky decomposition of the covariance matrices is used to orthogonalize the factors and obtained parameters in LG are not covariance matrices but Cholesky decomposed covariance matrices (Vermunt & Magidson, 2016).
