# Supplementary material for: Latent Markov Latent Trait Analysis for Exploring Measurement Model Changes in Intensive Longitudinal Data
Source: Eval Health Prof. 2020 Dec 11;44(1):61–76. doi: 10.1177/0163278720976762 (PMC7907986; doi:10.1177/0163278720976762)
Supplement: Supplemental Material, Accepted_VogelsmeierEtal2020_LMLTA_highlightedSupplement - Latent Markov Latent Trait Analysis for Exploring Measurement Model Changes in Intensive Longitudinal Data [file Accepted_VogelsmeierEtal2020_LMLTA_highlightedSupplement.pdf]

Online Supplement

S.1. Summary of the 3S Estimation

In this document, we provide a summary of the 3S estimation of the LMLTA model. In step 1, the state-specific MMs are obtained by estimating a mixture GPCM (e.g., Vermunt & Magidson, 2016). To this end, all repeated observations are treated as independent, which is valid because they are assumed to be conditionally independent given the state memberships at consecutive time-points. Hence, the relations between the states (i.e., the transitions) and the covariate effects that influence the transitions (as well as any latent class variable that clusters subjects by their transition patterns) are ignored in this step. The parameters of interest encompass (1) the state proportions, that is, the proportions of the observations that belong to each state, which is denoted as $p(s_k = 1)$, where s_k now refers to the state memberships across all subjects and time-points, and (2) the state-specific response probabilities $p(\mathbf{y}_{it}|s_{itk} = 1)$. The mixture GPCM is

$$p(\mathbf{y}_{it}) = \sum_{k=1}^{K} p(s_k = 1) p(\mathbf{y}_{it}|s_{itk} = 1) \tag{S1}$$

with $p(\mathbf{y}_{it}|s_{itk} = 1)$ as in Equation (9). The loglikelihood function is

$$\log\mathrm{L}_{STEP1} = \sum_{i=1}^{I} \sum_{t=1}^{T} \log p(\mathbf{y}_{it}). \tag{S2}$$

In order to find the ML estimates for the mixture model, LG combines an Expectation Maximization algorithm with Newton-Raphson iterations.

Subsequently, in step 2, we consider the posterior state probabilities, $p(s_{itk} = 1|\mathbf{y}_{it}) = p(s_k = 1)p(\mathbf{y}_{it}|s_{itk} = 1)/[\sum_{k'=1}^{K} p(s_{k'} = 1)p(\mathbf{y}_{it}|s_{itk'} = 1)]$, which are the probabilities for every subject and time-point to belong to each of the states. For every observation, we assign a state membership $p(w_{itm} = 1|\mathbf{y}_{it}) = 1$ to the state k with the highest posterior probability (i.e.,

the most likely state membership), which implies a weight of zero for all other states.[1] The indicators w_{itm} are collected in a new variable $\mathbf{w}_{it} = (w_{it1}, \ldots, w_{itK})'$ and, instead of the original observations $\mathbf{y}_{it}$, will be used for the estimation of the CT-LMM in step 3. As the highest posterior state probability is typically not equal to 1 for all observations, there will be classification error, which would lead to underestimation of the relation between the states and the covariates and the states at consecutive time-points if not accounted for in step 3. In order to calculate the errors we need to account for in step 3, we condition the assigned state memberships on the expected true state memberships $p(w_{itm} = 1 | s_{itk} = 1)$, for all $k, m = 1, \ldots, K$, and collect them in a $K \times K$ "classification error probability matrix". The entries of the matrix are calculated as (for details, see Di Mari et al., 2016; Vogelsmeier, Vermunt, Bülow, & De Roover, 2019):

$$p(w_{itm} = 1 | s_{itk} = 1) = \frac{\frac{1}{I \times T} \sum_{i=1}^{I} \sum_{t=1}^{T} p(w_{itm} = 1 | \mathbf{y}_{it}) p(s_{itk} = 1 | \mathbf{y}_{it})}{p(s_k = 1)}. \quad \text{(S3)}$$

Note that the diagonal elements (i.e., where $k = m$), correspond to the correctly classified observations and the off-diagonal elements to the classification errors.

Finally, in the third step, we estimate the (mixture) CT-LMM based on the state memberships that were determined in the previous step and correct for the inherent classification error. As was shown by Di Mari et al. (2016) and Vogelsmeier et al. (2019), this is done by treating the state assignments $\mathbf{w}_{it}$ as error-containing observed indicators of the error-free latent states $\mathbf{s}_{it}$ that are inferred through ML estimation and used to determine the parameters of the CT-LMM. To this end, the following loglikelihood with the classification-error probabilities $p(\mathbf{w}_{it} | \mathbf{s}_{it})$ as fixed response probabilities is maximized (Vogelsmeier et al., 2019):

[1] Note that this so-called "modal" assignment is the only feasible assignment procedure for a LMM with many subjects and time-points (Di Mari, Oberski, & Vermunt, 2016)

$$\log L_{STEP3} = \sum_{i=1}^{I} \log\left(\sum_{\mathbf{s}_{i1}} \cdots \sum_{\mathbf{s}_{iT}} p(\mathbf{s}_{i1}|\mathbf{z}_{i1}) \prod_{t=2}^{T} p_{\delta_{ti}}(\mathbf{s}_{it}|\mathbf{s}_{it-1}, \mathbf{z}_{it}) \prod_{t=1}^{T} p(\mathbf{w}_{it}|\mathbf{s}_{it})\right). \quad \text{(S4)}$$

Note that the loglikelihood of a mixture CT-LMM as used in the Application, where both the initial state and the transition probabilities may depend on a time-constant or time-varying latent class variable, has a slightly different form (e.g., Vermunt, Tran, & Magidson, 2008). In the simpler case of time-constant latent classes one gets:

$$\log L_{STEP3,mixture} = \quad \text{(S5)}$$

$$\sum_{i=1}^{I} \log\left(\sum_{\mathbf{c}_i} \sum_{\mathbf{s}_{i1}} \cdots \sum_{\mathbf{s}_{iT}} p(\mathbf{c}_i)\, p(\mathbf{s}_{i1}|\mathbf{z}_{i1}, \mathbf{c}_i) \prod_{t=2}^{T} p_{\delta_{ti}}(\mathbf{s}_{it}|\mathbf{s}_{it-1}, \mathbf{z}_{it}, \mathbf{c}_i) \prod_{t=1}^{T} p(\mathbf{w}_{it}|\mathbf{s}_{it})\right),$$

where $\mathbf{c}_i = (c_{i1}, \dots, c_{iV})'$ denotes the class memberships and $p(\mathbf{c}_i)$ the latent class or "mixture" proportions. LG obtains the ML parameter estimates by means of a combination of the forward-backward algorithm and the Newton-Raphson algorithm. For details on the mixture with a time-constant latent class variable, see Vermunt et al. (2008). The generalization to a mixture with a time-varying latent variable is straightforward and can be found in Crayen, Eid, Lischetzke, and Vermunt (2017).

S.2. Model Selection Procedure Step 1

In this document, we provide detailed information about the model selection procedure in step 1 of the 3S approach to estimate the LMLTA model. In order to see if the ML solutions of the nine estimated models were indeed global solutions, we estimated all models five times. The ML solutions were considered global solutions (at least, as far as we know) when the absolute difference between the solutions was smaller than 0.01. This was the case for the five one- and two-state models, but not for the three-state models. First, we compared the BIC values of the stable models. As can be seen from the BIC output below, the two-state model with two factors per state was the best (i.e. the model "[2 2]"), because it had the lowest BIC value.

Second, with the R-package "multichull", we conducted the CHull model selection procedure, which can be considered an automated scree test that identifies which models in a "loglikelihood versus number of parameters" figure are at the higher boundary of the convex hull (Cattell, 1966) and points out where the improvement in fit levels off when adding additional parameters (Bulteel, Wilderjans, Tuerlinckx, & Ceulemans, 2013; Ceulemans & Kiers, 2006; Ceulemans & Van Mechelen, 2005). Note that we also included the best ML solutions of the three-state models in the CHull procedure because the method entails that the most complex and most simple model cannot be chosen and the most complex model of the stable models would have been the best fitting model according to the BIC (i.e., the model [2 2]). However, sensitivity checks using all five local optima solutions revealed that the CHull would always come to the same conclusion. As can be seen from the CHull output below, the two best models were the one-state model with two factors (i.e., model [2] with a "scree test value" $st = 4.52$), and the two-state model with two factors in each state (i.e., model [2 2] with $st = 3.17$). Looking at the grouping of points that correspond to the different number of states in the convex hull figure below, it can be

seen that the improvement in fit is largest from one to two states, but that the improvement from two to three states is still substantial. For the application we chose the two-state model, [2 2], because it was among the best two models according to the CHull, better than the one-state model [2] according to the BIC, parameters differed considerably across the states (as is illustrated in the Application section of the main article), and finally, because it was well interpretable.

Output BIC.

```
      number of parameters BIC value
[1]                     84  300418.1
[2]                     95  286193.3
[1 1]                  169  273333.9
[2 1]                  180  263239.3
[2 2]                  191  259446.7
```

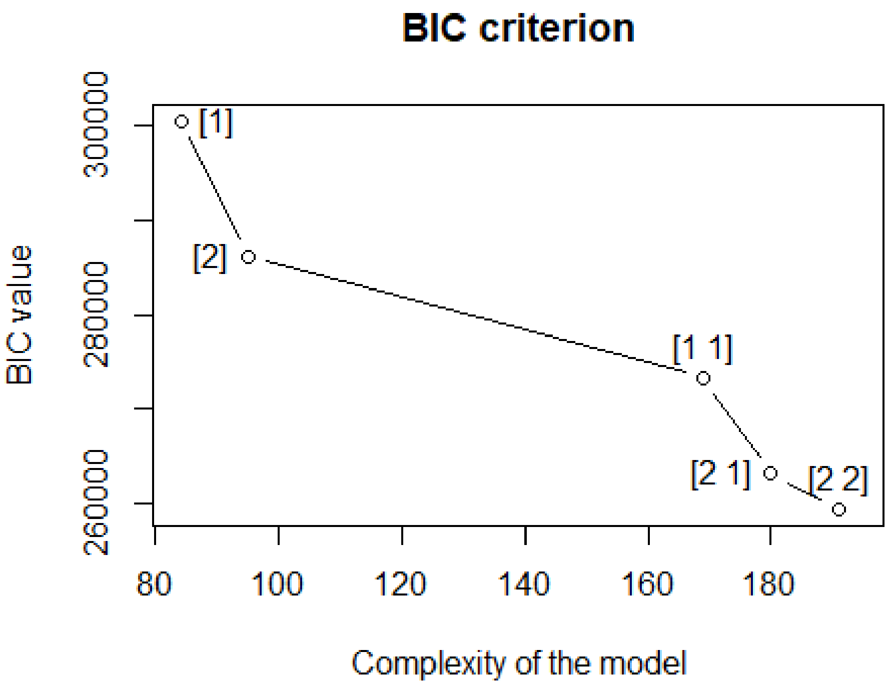

Output CHull.

```
SETTINGS BY USER:
Optimalization: upper bound
Required improvement in fit: 1%
Number of considered models: 9

RESULTS:
Number of selected models: 1

SELECTED MODEL:
    complexity       fit
[2]         95 -142641.7

ALL MODELS ON upper BOUND:
        complexity       fit       st
[1]             84 -149806.8       NA
[2]             95 -142641.7 4.520469
[2 2]          191 -128808.7 3.174246
[2 2 2]        287 -124450.8       NA


ORIGINAL MODELS
        complexity       fit
[1]             84 -149806.8
[2]             95 -142641.7
[1 1]          169 -135857.7
[2 1]          180 -130757.7
[2 2]          191 -128808.7
[1 1 1]        254 -129577.7
[2 1 1]        265 -127080.1
[2 2 1]        276 -125147.9
[2 2 2]        287 -124450.8
```

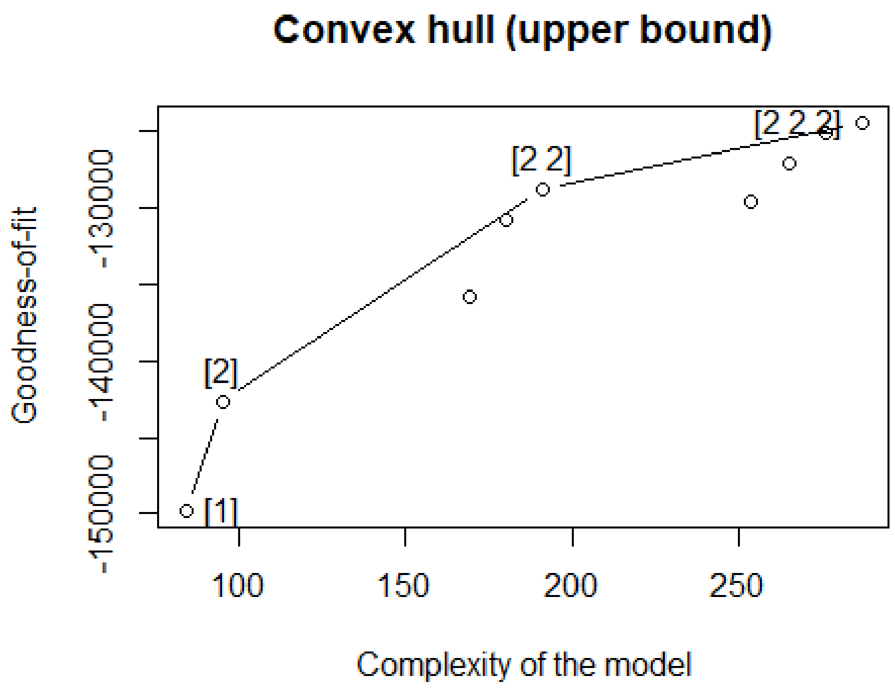

S.3. Additional Tables for the Application

In this document, we provide two additional tables related to the Application section. First, we investigated whether the state-specific MM patterns (i.e., that state 1 consists of the two factors PA and NA and that state 2 consists of the two factors HA-PA and PA/NA) only emerged as a consequence of constraining specific item loadings (i.e., setting the loadings of “happy” and “unhappy” equal to zero). To this end, we re-estimated the model [2 2] with factor covariance matrices $\mathbf{\Phi}_k$ set to identity matrices (i.e., uncorrelated factors that have variances of 1) and with the loadings of the item “irritated” set to equal to 0 for respectively the first factor. The resulting loadings are shown in the first table below, Supplement 3 Table 1. It can be seen that the first state again consists of the two factors PA and NA and the second state again consists of the two factors HA-PA and PA/NA. The only difference compared to the initial solution given in the Application (Table 1) is that, in state 2, the items “happy” and “excited” now have cross-loadings (i.e. they have large loadings on both factors) and not only on the first factor. Cross-loadings can be a result from constraining truly correlated factors to be uncorrelated. We conclude that the state-specific MM patterns emerge regardless of the chosen identification constrains.

Second, Supplement 3 Table 2, shows the differences in the $G - 1$ category intercept parameters for the 12 items across the two states. As can be seen, all intercepts differ significantly across the two states. In the Application, we investigate the between-state differences in the mean item scores (Table 1; an explanation of how to calculate the item means is provided in the same section). These differences directly follow from the intercept differences but are easier to interpret.

Supplement 3 Table 1

Differences in Item Means and Factor Loadings Across the two States with the Covariance Matrices Being Constrained to Identity Matrices

	State 1 loadings λ_{jr1}		State 2 loadings λ_{jr2}		Between-state loading difference statistics						Item means	
	$r = 1$	$r = 2$	$r = 1$	$r = 2$	$r = 1$			$r = 2$			State 1	State 2
Item j	PA	NA	HA-PA	PA/NA	Wald	df	p-value	Wald	df	p-value		
relaxed	**2.88**	-1.45	0.60	**-1.67**	20.01	1	< 0.01	0.61	1	0.43	5.72	6.89
content	**4.47**	-2.47	1.02	**-2.57**	15.13	1	< 0.01	0.04	1	0.84	5.76	6.92
confident	**2.29**	-1.22	0.59	**-1.29**	6.74	1	< 0.01	0.03	1	0.86	5.66	6.85
happy	**3.67**	-2.06	1.78	**-2.80**	19.53	1	< 0.01	6.08	1	0.01	5.62	6.81
energetic	**1.70**	-0.93	**1.94**	-1.74	0.27	1	0.60	5.05	1	0.02	5.21	6.41
excited	**2.28**	-1.28	2.29	**-2.34**	0.00	1	1.00	14.08	1	< 0.01	5.27	6.60
sad	-0.18	**3.31**	-0.34	**1.42**	2.56	1	0.11	13.88	1	< 0.01	1.09	1.03
unhappy	-0.34	**4.20**	-0.49	**1.78**	1.19	1	0.28	21.39	1	< 0.01	1.06	1.02
disappointed	-0.09	**4.26**	-0.31	**1.72**	3.13	1	0.08	22.16	1	< 0.01	1.07	1.04
angry	0.11	**3.81**	-0.25	**1.83**	17.64	1	< 0.01	25.09	1	< 0.01	1.04	1.02
nervous	-0.17	**1.80**	-0.09	**0.76**	1.12	1	0.29	6.38	1	0.01	1.24	1.09
irritated	<u>0.00</u>	**1.96**	<u>0.00</u>	**0.86**	/	/	/	8.54	1	< 0.01	1.24	1.16

Note. PA = Positive Affect; NA = Negative Affect; HA = High Arousal; LA = Low Arousal; j refers to items, and r to factors. For identification purposes, we set the underlined loadings of the item "irritated" on the first factors ($r = 1$) equal to 0. For each item and state, the loading with the largest absolute value is printed in boldface. Note that in state 2 the three items "happy", "energetic", and "excited" have high loadings on both factors.

Supplement 3 Table 2

Differences in the $G - 1$ Category Intercept Parameters ν_{jgk} for the 12 Items Across the two States

	State 1 intercepts ν_{jg1} for $g = (1, \ldots, 6)$						State 2 intercepts ν_{jg2} for $g = (1, \ldots, 6)$						Between-state difference statistics		
Item j	1	2	3	4	5	6	1	2	3	4	5	6	Wald	df	p-value
relaxed	-14.38	-6.97	-1.21	4.07	6.99	7.97	-8.24	-5.94	-2.43	1.23	3.08	5.01	284.20	6	< 0.01
content	-21.85	-10.89	-1.59	6.36	10.87	12.08	-13.16	-8.83	-3.41	1.71	5.04	8.06	224.20	6	< 0.01
confident	-9.63	-4.83	-0.68	2.76	4.81	5.71	-6.21	-5.09	-2.15	0.77	2.50	4.01	199.00	6	< 0.01
happy	-21.30	-10.01	-0.71	6.53	10.64	11.21	-10.8	-6.98	-2.83	1.78	4.51	6.27	295.50	6	< 0.01
energetic	-9.03	-3.54	0.35	3.76	5.05	4.70	-8.32	-5.48	-1.58	2.25	3.76	4.16	230.40	6	< 0.01
excited	-13.02	-5.02	0.39	5.03	6.95	6.52	-9.62	-6.45	-2.32	2.27	4.28	5.33	227.20	6	< 0.01
sad	17.79	15.5	10.34	4.34	-4.04	-12.87	8.27	4.63	2.51	0.56	-2.35	-5.58	131.70	6	< 0.01
unhappy	23.40	20.61	13.56	5.06	-5.51	-17.20	9.90	6.06	3.32	0.69	-2.91	-7.00	344.10	6	< 0.01
disappointed	22.34	19.77	13.13	4.89	-5.97	-18.77	9.24	5.72	3.25	0.54	-2.75	-6.42	61.47	6	< 0.01
angry	21.17	17.93	11.30	3.86	-6.04	-16.70	9.43	5.57	3.01	0.59	-2.62	-6.52	79.08	6	< 0.01
nervous	8.78	7.45	4.84	1.61	-2.46	-7.10	5.21	2.12	1.06	0.30	-1.32	-2.95	53.37	6	< 0.01
irritated	9.61	8.32	5.56	2.18	-2.79	-8.23	4.62	1.97	0.94	0.31	-1.30	-2.80	34.28	6	< 0.01

Note. j refers to items; g refers to the item categories; G refers to the number of item categories, which is 7 in the Grumpy or Depressed study.

S.4. Syntax for Running the Models

This document demonstrates the Latent GOLD (LG) syntax files to obtain the application results. Note that only one syntax is required for step 1 and 2 and one separate syntax is required for step 3.

Step 1 and 2 syntax. In this syntax, the regular ESM dataset is used as input. Note that all variables in the dataset that are not necessary in step 1 and 2 but that are necessary in step 3 (i.e., all the covariates, time-intervals, and subject IDs) have to be listed under “keep”. The variables are then added to the “classificationS1.csv” output file containing the posterior state probabilities. This is important because the file serves as only input for step 3 of the analysis as described next.

```
options
   algorithm tolerance=1e-008 emtolerance=0.01 emiterations=250
     nriterations=50;
   startvalues seed=0 sets=100 tolerance=1e-005 iterations=100;
   bayes latent=1 categorical=1;
   quadrature nodes=10;
   missing includeall;
output
   parameters=effect
   standarderrors
   profile
   estimatedvalues=model
   iterationdetails;

outfile
   'classificationS1.csv' classification
   keep ID deltaT depression family_c classmates_c friends_c NEWWAVE;

variables
  psuid ID ;
  dependent
     PA_LA1, PA_LA2, PA_LA3, PA_HA1, PA_HA2, PA_HA3, NA_LA1, NA_LA2,
     NA_LA3, NA_HA1, NA_HA2, NA_HA3;
  latent
      State nominal coding=first 2,
      F1 continuous,
      F2 continuous;

equations
      (c1)F1| State;
      (c2)F2| State;
```

```
      (c3)F1 <-> F2 | State;

      State <- 1 ;
      PA_LA1<- 1 | State + (a1)F1  | State + (b1)F2   | State;
      PA_LA2<- 1 | State + (a2)F1  | State + (b2)F2   | State;
      PA_LA3<- 1 | State + (a3)F1  | State + (b3)F2   | State;
      PA_HA1<- 1 | State + (a4)F1  | State + (b4)F2   | State;
      PA_HA2<- 1 | State + (a5)F1  | State + (b5)F2   | State;
      PA_HA3<- 1 | State + (a6)F1  | State + (b6)F2   | State;
      NA_LA1<- 1 | State + (a7)F1  | State + (b7)F2   | State;
      NA_LA2<- 1 | State + (a8)F1  | State + (b8)F2   | State;
      NA_LA3<- 1 | State + (a9)F1  | State + (b9)F2   | State;
      NA_HA1<- 1 | State + (a10)F1 | State + (b10)F2  | State;
      NA_HA2<- 1 | State + (a11)F1 | State + (b11)F2  | State;
      NA_HA3<- 1 | State + (a12)F1 | State + (b12)F2  | State;

  //Constraints (on "I feel happy/unhappy")
      a4[1,]=1;
      a8[1,]=0;
      b4[1,]=0;
      b8[1,]=1;
      a4[2,]=1;
      a8[2,]=0;
      b4[2,]=0;
      b8[2,]=1;
```

Step 3 syntax full model. In this syntax, the “classificationS1.csv” file is used as input. When using LG’s “step3” option, the software automatically calculates the classification error probability matrix from the posterior state probabilities. In order to specify the columns of the classificationS1.csv file in which LG can find the posterior probabilities, the user has to provide the column names as “posterior = (State.1 State.2)”. Note that the column names depend on the name that was used to define the state variable in the step 1 and 2 syntax. Also note that we used a CT-LMM for the latent states and a DT-LMM for the latent classes. While subjects were allowed to transition between the states with every new observation, subjects were allowed to transition between the classes only at the beginning of every new wave. Therefore, we added a variable (“NEWWAVE”) that indicated whether a record concerned a new wave (NEWWAVE = 1) or whether a record was another observation from the same wave (NEWWAVE = 0). By means of

constraints on the logits, all transition probabilities for NEWWAVE = 0 were set to zero. Moreover, 19 subjects skipped wave 2. If this was ignored, LG would assume that all intervals between the waves were the same (i.e., approximately 3 months) although there were 19 longer intervals (i.e., approximately 6 months), which could lead to inaccurate parameter estimates. To solve this problem, 19 empty records (i.e., with missing values on all variables but the ID and the NEWWAVE variable) were added to the "classificationS1.csv" file. By choosing to including all records with missing observation ("missing includeall"), LG accounts for the fact that that the second wave has been skipped and corrects for this when estimating the transition probabilities. Finally, note that the final latent state assignments may differ from the initial state assignments (i.e., the single indicators) when the classification error is rather large. In order to see the final state assignments, the user has to add the command "noignoreclassification" to the "step3" option.

```
options
  algorithm tolerance=1e-008 emtolerance=0.01 emiterations=250
    nriterations=50 expm=pade;
  startvalues seed=0 sets=10 tolerance=1e-005 iterations=100;
  bayes latent=1 categorical=1 ct=1;
  missing includeall;
  step3 ml modal noignoreclassification;

output
  parameters=effect
  standarderrors
  profile
  iterationdetails
  estimatedvalues=model
  classification;

variables
  caseid ID;
  independent family_c nominal coding=first, classmates_c nominal
    coding=first, friends_c nominal coding=first, NEWWAVE nominal
    coding=first, depression2 nominal coding=first;
  timeinterval deltaT;

  latent State nominal dynamic posterior=(State.1 State.2)
coding=first, Class
    nominal dynamic dt 3 coding=first;
```

```
equations
   Class[=0] <- 1 + depression;
   Class <- (b~tra) 1 | Class[-1] NEWWAVE
                      + (~tra) depression| Class[-1];

   State[=0] <- 1;
   State <- (~tra) 1 | State[-1]
                      + (~tra) Class | State[-1]
                      + (~tra) family_c  | State[-1] Class
                      + (~tra) classmates_c   | State[-1] Class
                      + (~tra) friends_c | State[-1] Class;

   b[1] = -100;
   b[2] = -100;
   b[3] = -100;
```

Step 3 syntax reduced model. This syntax is the same as the step 3 syntax for the full model but without the covariate effects of depression on the initial class and class transition probabilities and without the effect of being with friends on the state-transitions conditional on the class. Instead, the unconditional effect of being with friends on the state-transitions was added. Below, we only stated the changed equations.

```
equations
   Class[=0] <- 1;
   Class <- (b~tra) 1 | Class[-1] NEWWAVE;

   State[=0] <- 1;
   State <- (~tra) 1 | State[-1]
                      + (~tra) Class | State[-1]
                      + (~tra) family_c  | State[-1] Class
                      + (~tra) classmates_c   | State[-1] Class
                      + (~tra) friends_c | State[-1];

   b[1] = -100;
   b[2] = -100;
   b[3] = -100;
```

S.5. Model Selection Procedure Step 3

In this document, we provide information about the model selection procedure that was used to determine the number of latent classes in step 3 of the estimation (note that a description of the CHull procedure is provided in the Online Supplement S.2). In contrast to the model selection in step 1 (Online Supplement S.2), the models in step 3 were estimated only once because local maxima are very unlikely when the MMs are fixed. First, we estimated the full model ("F") as specified in Equation (11) with 1–3 classes. Investigating the models with 2 and 3 classes, we saw that depression did neither predict the initial state probabilities nor the transition probabilities for the classes. Furthermore, the effect of being with friends on the transition intensities for the states appeared to be significant but did not significantly differ across classes. Since it was already apparent form the BIC that the full 3-class model fitted better than the 1- and 2-class models, we also examined a 4-class model in order not to overlook a relevant class. However, the full model with 4 classes did not converge and was therefore not considered in the model selection procedure.

Subsequently, we re-estimated the models with multiple classes (including the 4-class model), leaving out the effects that were non-significant in the full models (i.e., the effect of depression on the initial class and transition probabilities between classes and the effect of being with friends depending on the class), while including the unconditional effect of being with friends (i.e., not conditional on the class). All reduced ("R") models converged. As can be seen from the BIC and CHull outputs below, the reduced model with 3 classes (i.e., the model "R3classes") had the best fit according to the BIC, as it has the lowest BIC value, and was under the best three models according to the CHull when considering all converged full and reduced models.

Although we chose the reduced model with three classes, we also investigated the reduced model with two classes as the improve in fit when adding a third class was rather small (as can be

seen from the BIC and CHull plots). Similar to the three-class solution, the two-class solution had one stable class. In the other class, adolescents had a high probability to transition between to states with a slightly higher probability to move to and stay in state 2. Therefore, if we would have considered the 2-class solution, we would have missed the third class, in which adolescents frequently transition between the states but are more likely to transition to and stay in state 1.

Output BIC.

```
           number of parameters BIC value
F1class                       9  13677.23
R2classes                    18  12056.45
F2classes                    23  12097.09
R3classes                    29  11828.40
F3classes                    41  11923.62
R4classes                    42  11847.99
```

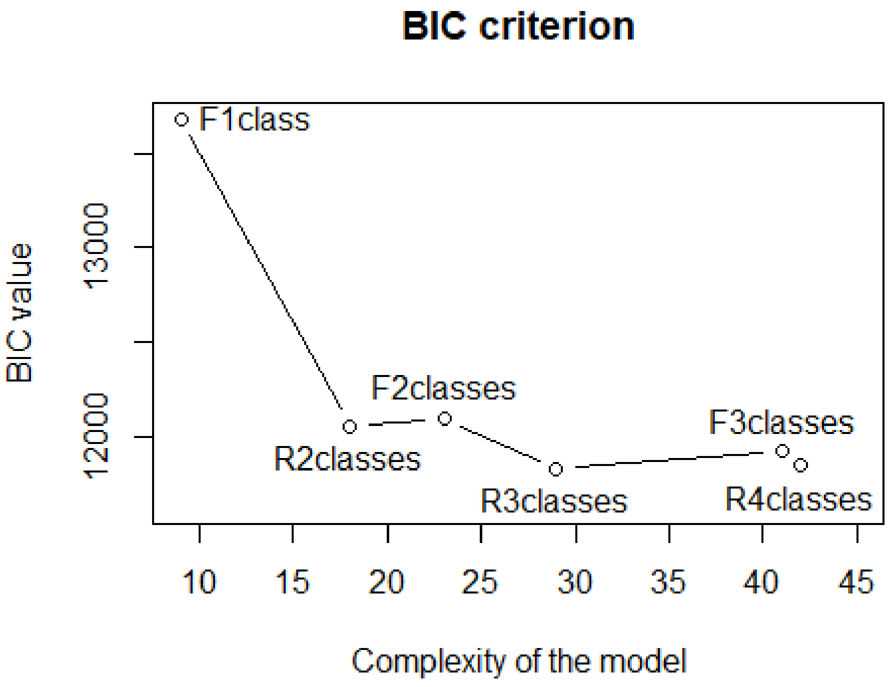

Output CHull.

```
SETTINGS BY USER:
Optimalization: upper bound
Required improvement in fit: 1%
Number of considered models: 6

RESULTS:
Number of selected models: 1

SELECTED MODEL:
          complexity       fit
R2classes         18 -5942.029

ALL MODELS ON upper BOUND:
          complexity       fit       st
F1class            9 -6795.519       NA
R2classes         18 -5942.029 6.257659
R3classes         29 -5775.329       NA


ORIGINAL MODELS
          complexity       fit
F1class            9 -6795.519
R2classes         18 -5942.029
F2classes         23 -5938.405
R3classes         29 -5775.329
F3classes         41 -5765.476
R4classes         42 -5722.873
```

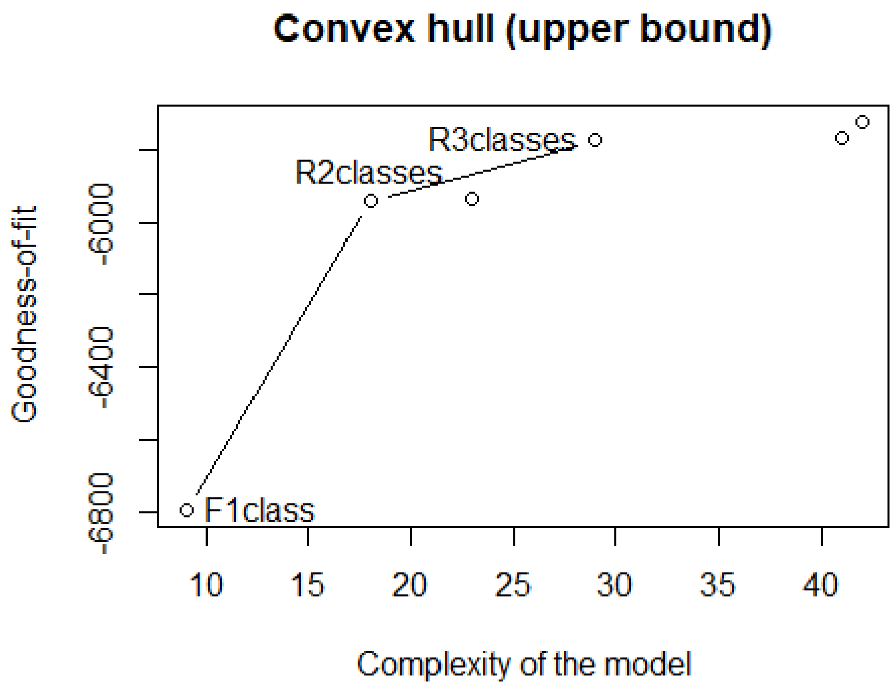

S.6. R Code to Calculate Transition Probabilities

In this document, we show how to calculate transition probabilities between the states for a given class membership and covariate value and for any interval of interest. More specifically, we calculate the transition probability matrix for being with family in class 2 and a median interval length (i.e., 2.25 hours). As explained in the Latent Markov Latent Trait Analysis section, the log intensities can be calculated as $\log q_{lk} = \gamma_{0lk} + \boldsymbol{\gamma}'_{lk}\mathbf{z}_{it}$ and the transition probabilities $\mathbf{P}_{2.25}$ are the matrix exponential of $\mathbf{Q} \times 2.25$.

```
library(expm)

# fill the estimates from table 2 into equation (12) from the article.
# note that the 0's and 1's are the values on the dummy variables.
# for example, -0.63 * 0 implies that we calculate the
# transition intensity when not being with friends.

q12 <- -0.55 +      # transition intercept state
        0.00 * 1 +  # effect of class for class 2
       -7.21 * 0 +  # effect of class for class 3
       -0.48 * 0 +  # effect of family x class 1
       -0.10 * 1 +  # effect of family x class 2
       -1.11 * 0 +  # effect of family x class 3
       -2.62 * 0 +  # effect of classmates x class 1
       -0.75 * 0 +  # effect of classmates x class 2
       -2.70 * 0 +  # effect of classmates x class 3
       -0.63 * 0    # effect of friends

q21 <- -0.08 +      # transition intercept state
       -1.71 * 1 +  # effect of class for class 2
       -8.74 * 0 +  # effect of class for class 3
       -0.63 * 0 +  # effect of family x class 1
       -1.12 * 1 +  # effect of family x class 2
       -2.27 * 0 +  # effect of family x class 3
       -1.30 * 0 +  # effect of classmates x class 1
        0.51 * 0 +  # effect of classmates x class 2
       -0.96 * 0 +  # effect of classmates x class 3
       -0.39 * 0    # effect of friends

# put log intensities in a matrix:
LogIntensities <- matrix(c(0, q12,
                           q21, 0),
                         nrow = 2, ncol = 2, byrow = TRUE)

# exponentiate to obtain intensities:
```

```
Intensities <- exp(LogIntensities)

# at this point, we already have the intensities of the
# off-diagonal elements. the diagonal elements are equal to
# the negative row sums of the off-diagonal elements:
for (i in 1:ncol(Intensities)) {
  Intensities[i, i] <- -(sum(Intensities[i, -i]))
}
# get the probabilities for the median interval d = 2.25
# (note that we can specify any interval of interest here):
d <- 2.25
TransitionProbabilities <- expm(Intensities * d)

# transition probabilities when being with family (in class 2).
# as can be seen, there is a high probability to transition to
# state 2 (66%) and to stay in state 2 (93%):
round(TransitionProbabilities, digits = 2)

##      [,1] [,2]
## [1,] 0.34 0.66
## [2,] 0.07 0.93
```

References Supplement

Bulteel, K., Wilderjans, T. F., Tuerlinckx, F., & Ceulemans, E. (2013). CHull as an alternative to AIC and BIC in the context of mixtures of factor analyzers. *Behavior Research Methods, 45*, 782–791. doi:10.3758/s13428-012-0293-y

Cattell, R. B. (1966). The scree test for the number of factors. *Multivariate Behavioral Research, 1,* 245–276. doi:10.1207/s15327906mbr0102_10

Ceulemans, E., & Kiers, H. A. (2006). Selecting among three-mode principal component models of different types and complexities: a numerical convex hull based method. *British Journal of Mathematical and Statistical Psychology, 59,* 133–150. doi:10.1348/000711005X64817

Ceulemans, E., & Van Mechelen, I. (2005). Hierarchical classes models for three-way three-mode binary data: interrelations and model selection. *Psychometrika, 70*, 461–480. doi:10.1007/s11336-003-1067-3

Crayen, C., Eid, M., Lischetzke, T., & Vermunt, J. K. (2017). A continuous-time mixture latent-state-trait Markov model for experience sampling data. *European Journal of Psychological Assessment, 33*, 296–311. doi:10.1027/1015-5759/a000418

Di Mari, R., Oberski, D. L., & Vermunt, J. K. (2016). Bias-adjusted three-step latent Markov modeling with covariates. *Structural Equation Modeling: A Multidisciplinary Journal, 23*, 649–660. doi:10.1080/10705511.2016.1191015

Vermunt, J. K., & Magidson, J. (2016). *Technical Guide for Latent GOLD 5.1: Basic, Advanced, and Syntax*. Belmont, MA: Statistical Innovations.

Vermunt, J. K., Tran, B., & Magidson, J. (2008). Latent class models in longitudinal research. In S. Menard (Ed.), *Handbook of Longitudinal Research: Design, Measurement, and Analysis* (pp. 373–385). Burlington, MA: Elsevier.

Vogelsmeier, L. V. D. E., Vermunt, J. K., Bülow, A., & De Roover, K. (2019). *Evaluating covariate effects on ESM measurement model changes with latent Markov factor analysis: A three-step approach*. Preprint. Retrieved from https://doi.org/10.31234/osf.io/6ufrc
